# RAId_DbS: Peptide Identification using Database Searches with Realistic Statistics

**DOI:** 10.1186/1745-6150-2-25

**Published:** 2007-10-25

**Authors:** Gelio Alves, Aleksey Y Ogurtsov, Yi-Kuo Yu

**Affiliations:** 1 National Center for Biotechnology Information, National Library of Medicine, NIH, Bethesda, MD 20894

## Abstract

**Background:**

The key to mass-spectrometry-based proteomics is peptide identification. A major challenge in peptide identification is to obtain realistic *E*-values when assigning statistical significance to candidate peptides.

**Results:**

Using a simple scoring scheme, we propose a database search method with theoretically characterized statistics. Taking into account possible skewness in the random variable distribution and the effect of finite sampling, we provide a theoretical derivation for the tail of the score distribution. For every experimental spectrum examined, we collect the scores of peptides in the database, and find good agreement between the collected score statistics and our theoretical distribution. Using Student's *t*-tests, we quantify the degree of agreement between the theoretical distribution and the score statistics collected. The T-tests may be used to measure the reliability of reported statistics. When combined with reported *P*-value for a peptide hit using a score distribution model, this new measure prevents exaggerated statistics. Another feature of RAId_DbS is its capability of detecting multiple co-eluted peptides. The peptide identification performance and statistical accuracy of RAId_DbS are assessed and compared with several other search tools. The executables and data related to RAId_DbS are freely available upon request.

## Open peer review

Reviewed by Frank Eisenhaber, Wing-Cheong Wong (co-reviewer invited by Frank Eisenhaber), Dongxiao Zhu (nominated by Arcady Mushegian) and Shamil Sunyaev. For the full reviews, please go to the Reviewers' comments section.

## Introduction

Protein identification is the key to proteomics. As an indispensable component in mass spectrometry (MS) based protein identification, peptide identification through tandem MS (MS^2^) is usually aided by automated data analysis. Among available data analysis tools, methods based on database searches are most frequently used. Methods using database searches may be roughly classified into two categories, depending on whether or not they provide *E*-values (or *P*-values) for candidate peptides. Methods – using either correlation, posterior probabilities, score, or Z-score – include, but are not limited to, SEQUEST [1], MS-Tag [2], Scope [3], CIDentify [4], Popitam [5], ProbID [6], and PepSearch [7]. Examples of database search methods directly reporting *P*- or *E*-values include, but are not limited to, Mascot [8], Sonar [9], InsPecT [10], OMSSA [11], and X!Tandem [12]. A comprehensive survey may be found in [13] and a performance evaluation of several of the methods mentioned can be found in [14].

For a given quality score cutoff S, *E*-value is defined as the expected number of hits, in a random database, with quality score being the same as or higher than the cutoff. (Similarly, *P*-value refers to the probability of finding a random hit with quality score being the same as or higher than the cutoff.) A realistic *E*-value assignment thus provides the user with the number of false positives to anticipate. Our goal in developing RAId_DbS (Robust Accurate Identification of Peptides in Database Search) is to provide a database search method with realistic *E*-value assignments. Among methods that report *E*-values, we find the approach employed by [15] is closest to what we have developed. Basically, both methods use the *real *score histogram from scoring database peptides against a query spectrum to form the basis of score statistics. The difference, however, lies in the fact RAId_DbS has its score statistics founded on a theoretical distribution, while the method of [15] assumes an exponential distribution pdf(S) ≈ exp(-λS) for large score S.

To illustrate an important aspect of peptide score statistics, let us note that the noise in an MS^2 ^spectrum is spectrum-specific. That is, it is not yet possible to predict spectral noise, which nonetheless influences the scoring of candidate peptides for a given spectrum. One of our goals in developing RAId_DbS is to take into account the fact that noise is spectrum-specific. This goal is achieved by using a scoring scheme whose statistics can be theoretically characterized. Our scoring scheme is largely similar to that proposed by [16]. However, in addition to the introduction of weight factors to encourage mass accuracy, we have taken into account the effect of finite sampling and finite skewness and have derived a *new *score distribution function replacing the Gaussian distribution assumed by [16].

Typical database search methods usually ask the user to set a maximum number of allowed enzymatic miscleavages. Not only do we lift this constraint, we even allow for non-canonical N-terminal cleavages (NNTC), also referred to as "incorrect N-terminal cleavages" by [17]. These are handled efficiently by first scoring *exhaustively *all the four-letter C- and N-terminal tags to produce two high-scoring tag lists, one for each terminal. A candidate peptide with NNTC will be scored only when one of its two terminal tags ranks high enough in its respective list.

Unless otherwise mentioned, we limit our discussions to methods that directly report *E*-values (or *P*-values). This is because converting scores, correlation coefficients, *etc*. into *E*-values is non-trivial and is method-dependent. In fact, even converting *E*-values reported by one method to *E*-values reported by other method is already non-trivial. This important task of standardizing *E*-values, although beyond the scope of the current paper, will be addressed in a forthcoming publication [18].

To better highlight the main points of this paper, we have relegated to the appendix details such as m/z peak filtering, tag scoring, and a detailed description of implementation. Throughout the paper, we use the dalton (Da) as the unit for molecular weight. In the following, we first provide a brief description of the two different types of data (centroid and profile) used, and the experimental protocol used to obtain the profile spectra. We will then describe RAId_DbS's scoring scheme, followed by a detailed description of the mathematical underpinning of the score statistics. The *E*-value test and the performance test will then be described followed by a section discussing the importance of quantifying the goodness of score distribution modeling in statistical inference. We conclude in the last section with some relevant remarks.

## Experiment

Two data sets were used in this study. The centroid mode data set developed by the Institute for Systems Biology [19] was used only for performance comparison, while the profile mode data set was used for both statistical assessment and performance comparison. Because RAId_DbS is designed to take profile mode data and most of the published data are collected in centroid mode, it was necessary to generate profile data for this study. The profile mode data we used was provided by Dr. R.-F. Shen, the director of the mass spectrometry core facility at the National Heart, Lung, and Blood Institute (NHLBI). The acquisition of those profile mode spectra is described below.

A mixture of 7 proteins (Sigma) containing equimolar levels of *α*-lactalbumin (LALBA_BOVIN, P00711), lysozyme (LYSC_CHICK, P00698), *β*-lactoglobulin B (LACB_BOVIN, P02754), hemoglobin (HBA_HUMAN and HBB_HUMAN, P69905 and P68871), bovine serum albumin (ALBU_BOVIN, P02769), apotransferrin (TRFE_HUMAN, P02787), and *β*-galactosidase (BGAL_ECOLI, P00722) was used for all experiments. Note that both the *α *chain and the *β *chain of hemoglobin are included. The protein mixture in 50 mM ammonium bicarbonate buffer was reduced with 10 mM DTT at 60°C for 1 hr, alkylated with 55 mM iodoacetamide at room temperature in the dark for 30 min, and digested with trypsin (Promega) at 50:1 mass ratio at 37°C overnight, as described in [20]. Three different levels of protein mixture (50 fmols, 500 fmols, and 5 pmols of each protein) were then injected into LC/MS/MS. Two different kinds of mass spectrometers were utilized in this study, nanospray (NSI)/LTQ FT (Thermo Finnigan) and matrix assisted laser desorption ionization (MALDI)/TOF/TOF (Applied Biosystems). For NSI/LTQ FT, following [21], peptides were first loaded onto a trap cartridge (Agilent) at a flow rate of 2 *μ*l/min. Trapped peptides were then eluted onto a reversed-phase PicoFrit column (New Objective) using a linear gradient of acetonitrile (0–60%) containing 0.1% FA. The duration of the gradient was 20 min at a flow rate of 0.25 *μ*l/min, which was followed by 80% acetonitrile washing for 5 minutes. The eluted peptides from the PicoFrit column were nano-sprayed into an LTQ FT mass spectrometer. The data-dependent acquisition mode was enabled, and each survey MS scan was followed by five MS/MS scans with the dynamic exclusion option on. The spray voltage and ion transfer tube temperature were set at 1.8 kV and 160°C, respectively. The normalized collision energy was set at 35%. Three different combinations of mass analyzers (LTQ LTQ, LTQ FT, and FT FT) were used to acquire protein mixtures at each level. For MALDI/TOF/TOF, following [22], peptide separation was performed on a Famos/Switchos/Ultimate chromatography system (Dionex/LC Packings) equipped with a Probot (MALDI-plate spotting device). Peptides were injected and captured onto a trap column (PepMap C18, 5 *μ*m, 100 A, 300 *μ*m i.d. × 5 mm) at 10 *μ*l/min. Peptide separation was achieved on an analytical nano-column (PepMap C18, 3 *μ*m, 100 A, 75 *μ*m i.d. × 15 cm) using a gradient of 5 to 60% solvent B in A over 90 min (solvent A: 100% water, 0.1% TFA; solvent B: 80% acetonitrile/20% water, 0.1% TFA), 60 to 95% solvent B in A for 1 min, and then 95% solvent B for 19 min at a flow rate of 0.16 *μ*l/min. The HPLC eluant was supplemented with 5 mg/ml *α*-cyano-4-hydroxycinnamic acid (in 50/50 acetonitrile/water containing 0.1% TFA) from a syringe pump at a flow rate of 1 *μ*l/min, and spotted directly onto the ABI 4700 576-well target plates using the Probot. MALDI/TOF/TOF data were acquired in batch mode.

## Scoring scheme

Like many other peptide analysis methods, RAId_DbS uses primarily *b*- and *y*-series peaks to score a candidate peptide or a tag. As will be seen, it is simple to include more evidence peaks, and it is also straightforward to switch to a different peak series for scoring. For example, in place of *b*- and *y*-series, one may use *c*- and *z*-series for scoring. This will be useful for analyzing spectra generated by the electron transfer dissociation (ETD) method [23].

The score of a peptide π is given as

S(π)=1T(π)∑i∈{b(π)∪y(π)}T(π)wi(mi)ln⁡[ℐ(i)],
 MathType@MTEF@5@5@+=feaafiart1ev1aaatCvAUfKttLearuWrP9MDH5MBPbIqV92AaeXatLxBI9gBaebbnrfifHhDYfgasaacH8akY=wiFfYdH8Gipec8Eeeu0xXdbba9frFj0=OqFfea0dXdd9vqai=hGuQ8kuc9pgc9s8qqaq=dirpe0xb9q8qiLsFr0=vr0=vr0dc8meaabaqaciaacaGaaeqabaqabeGadaaakeaacqqGtbWucqGGOaakiiGacqWFapaCcqGGPaqkcqGH9aqpdaWcaaqaaiabigdaXaqaaiabdsfaujabcIcaOiab=b8aWjabcMcaPaaadaaeWbqaaiabdEha3naaBaaaleaacqWGPbqAaeqaaOGaeiikaGIaemyBa02aaSbaaSqaaiabdMgaPbqabaGccqGGPaqkcyGGSbaBcqGGUbGBcqGGBbWwt0uy0HwzTfgDPnwy1egaryqtHrhAL1wy0L2yHvdaiqaacqGFqesscqGGOaakcqWGPbqAcqGGPaqkcqGGDbqxaSqaaiabdMgaPjabgIGiolabcUha7jabdkgaIjabcIcaOiab=b8aWjabcMcaPiabgQIiilabdMha5jabcIcaOiab=b8aWjabcMcaPiabc2ha9bqaaiabdsfaujabcIcaOiab=b8aWjabcMcaPaqdcqGHris5aOGaeiilaWcaaa@6B8E@

where *b*(*π*)(*y*(*π*)) represents the set of theoretical *b *(*y*) peaks of peptide π, *T*(π) is the total number of peaks when one unites *b*(π) and *y*(π), ℐ
 MathType@MTEF@5@5@+=feaafiart1ev1aaatCvAUfKttLearuWrP9MDH5MBPbIqV92AaeXatLxBI9gBaebbnrfifHhDYfgasaacH8akY=wiFfYdH8Gipec8Eeeu0xXdbba9frFj0=OqFfea0dXdd9vqai=hGuQ8kuc9pgc9s8qqaq=dirpe0xb9q8qiLsFr0=vr0=vr0dc8meaabaqaciaacaGaaeqabaqabeGadaaakeaat0uy0HwzTfgDPnwy1egaryqtHrhAL1wy0L2yHvdaiqaacqWFqessaaa@3768@(*i*) = max{*I*_*i*_, 1} with *I*_*i *_being the intensity found in the *processed *query spectrum (see appendix) for the peak labeled *i *with m/z value *m*_*i *_in the set {*b*(π) ∪ *y *(π)}. The weight factor *w*_*i *_is introduced to emphasize peaks with less mass error. The default is *w*_*i*_(*m*_*i*_) = exp(*-|Δm*_*i*_|) with Δ*m*_*i *_= *m*_*i *_- *t*_*i *_being the difference between the observed m/z value *m*_*i *_and the theoretical value *t*_*i*_.

In the absence of weighting, our scoring scheme is the same as that of [16]. The difference between our method and that proposed by [16] lies in the theoretical distribution being derived as opposed to assumed. When *w*_*i *_= 1, one will pick the strongest intensity in range *i *([*t*_*i *_- 1, *t*_*i *_+ 1]) as *I*_*i*_. With weighting, RAId_DbS first multiplies the log intensity of each candidate peak in the mass range *i *by *w*_*i*_(*m*_*i*_) and then pick the maximum *w*_*i*_(*m*_*i*_) ln [ℐ
 MathType@MTEF@5@5@+=feaafiart1ev1aaatCvAUfKttLearuWrP9MDH5MBPbIqV92AaeXatLxBI9gBaebbnrfifHhDYfgasaacH8akY=wiFfYdH8Gipec8Eeeu0xXdbba9frFj0=OqFfea0dXdd9vqai=hGuQ8kuc9pgc9s8qqaq=dirpe0xb9q8qiLsFr0=vr0=vr0dc8meaabaqaciaacaGaaeqabaqabeGadaaakeaat0uy0HwzTfgDPnwy1egaryqtHrhAL1wy0L2yHvdaiqaacqWFqessaaa@3768@(*i*)] from each of the mass ranges {[ti−1,ti+1]}i=1T(π)
 MathType@MTEF@5@5@+=feaafiart1ev1aaatCvAUfKttLearuWrP9MDH5MBPbIqV92AaeXatLxBI9gBaebbnrfifHhDYfgasaacH8akY=wiFfYdH8Gipec8Eeeu0xXdbba9frFj0=OqFfea0dXdd9vqai=hGuQ8kuc9pgc9s8qqaq=dirpe0xb9q8qiLsFr0=vr0=vr0dc8meaabaqaciaacaGaaeqabaqabeGadaaakeaacqGG7bWEcqGGBbWwcqWG0baDdaWgaaWcbaGaemyAaKgabeaakiabgkHiTiabigdaXiabcYcaSiabdsha0naaBaaaleaacqWGPbqAaeqaaOGaey4kaSIaeGymaeJaeiyxa0LaeiyFa03aa0baaSqaaiabdMgaPjabg2da9iabigdaXaqaaiabdsfaujabcIcaOGGaciab=b8aWjabcMcaPaaaaaa@44E4@. The same scoring method is used for sequence tags.

When the number *T*(π) in Eq. (1) is fixed and large, one would anticipate the central limit theorem to hold and the score distribution be a Gaussian. However, for a typical search *T*(π) is not fixed and not necessarily large enough to guarantee that the skewness is negligible. In the next section, we will derive a new probability distribution function, whose end results is given in Eq. (17), to accommodate the finite sampling and skewness. It turns out that even with the weights included to encourage better m/z matching, the score statistics of Eq. (1) still follow the general form given by Eq. (17).

When the spectrum contains little information, it becomes inappropriate to use Eq. (17). The *P*-value of a candidate peptide of *L *amino acids and with weighted peak count ∑i∈{b(π)∪y(π)}T(π)wi(mi)≡c
 MathType@MTEF@5@5@+=feaafiart1ev1aaatCvAUfKttLearuWrP9MDH5MBPbIqV92AaeXatLxBI9gBaebbnrfifHhDYfgasaacH8akY=wiFfYdH8Gipec8Eeeu0xXdbba9frFj0=OqFfea0dXdd9vqai=hGuQ8kuc9pgc9s8qqaq=dirpe0xb9q8qiLsFr0=vr0=vr0dc8meaabaqaciaacaGaaeqabaqabeGadaaakeaadaaeWaqaaiabdEha3naaBaaaleaacqWGPbqAaeqaaOGaeiikaGIaemyBa02aaSbaaSqaaiabdMgaPbqabaGccqGGPaqkcqGHHjIUcqqGJbWyaSqaaiabdMgaPjabgIGiolabcUha7jabdkgaIjabcIcaOGGaciab=b8aWjabcMcaPiabgQIiilabdMha5jabcIcaOiab=b8aWjabcMcaPiabc2ha9bqaaiabdsfaujabcIcaOiab=b8aWjabcMcaPaqdcqGHris5aaaa@4F34@ is then estimated by the following heuristic formula

P=∑j=[c]+12(L−1)(2L−2)!j!(2L−2−j)! pj(1−p)2L−2−j,
 MathType@MTEF@5@5@+=feaafiart1ev1aaatCvAUfKttLearuWrP9MDH5MBPbIqV92AaeXatLxBI9gBaebbnrfifHhDYfgasaacH8akY=wiFfYdH8Gipec8Eeeu0xXdbba9frFj0=OqFfea0dXdd9vqai=hGuQ8kuc9pgc9s8qqaq=dirpe0xb9q8qiLsFr0=vr0=vr0dc8meaabaqaciaacaGaaeqabaqabeGadaaakeaacqWGqbaucqGH9aqpdaaeWbqaamaalaaabaGaeiikaGIaeGOmaiJaemitaWKaeyOeI0IaeGOmaiJaeiykaKIaeiyiaecabaGaemOAaOMaeiyiaeIaeiikaGIaeGOmaiJaemitaWKaeyOeI0IaeGOmaiJaeyOeI0IaemOAaOMaeiykaKIaeiyiaecaaaWcbaGaemOAaOMaeyypa0Jaei4waSLaee4yamMaeiyxa0Laey4kaSIaeGymaedabaGaeGOmaiJaeiikaGIaemitaWKaeyOeI0IaeGymaeJaeiykaKcaniabggHiLdGccqqGGaaicqWGWbaCdaahaaWcbeqaaiabdQgaQbaakiabcIcaOiabigdaXiabgkHiTiabdchaWjabcMcaPmaaCaaaleqabaGaeGOmaiJaemitaWKaeyOeI0IaeGOmaiJaeyOeI0IaemOAaOgaaOGaeiilaWcaaa@6037@

where [c] represents the integer part of c *> *0, *p *≡ ⟨c⟩/(*L*_eff_) with ⟨c⟩ representing c averaged over all peptides entering the final scoring, and *L*_eff _≡ Molecular weight/110Da. Formula (2), heuristic in nature, is invoked if ⟨c⟩ ≤ 2 to provide a conservative *E*-value. There is apparently room for improvement in scoring spectrum with little information.

## Theory

In this section, we will provide a heuristic derivation to solve a generic problem that may occur in various scientific fields. Specifically, we will address how the Gaussian distribution assured by the central limit theorem can be modified in the presence of skewness and finite sampling. To deal with skewness and finite sampling size is by no means a new front of attack. There exist many well written literatures [24,25] touching upon this subject. However, the deviations (due to finite size and/or skewness) from the Gaussian distribution are usually dealt with through computing the difference between the pre-Gaussian and the Gaussian using Hermite polynomials [24,25]. Adding only a few or finite number of those correction terms sometimes roughens the tail of the distribution function. We provide a different way to derive the distribution function, incorporating the finite size effect and the skewness, that has a smooth tail and has the correct asymptotics in a closed form.

The random variable *x *corresponds to the logarithm of m/z peak intensity whose distribution *g*(*x*) is governed by the experimental spectrum under consideration. Because of the use of a logarithm, a rescaling of peak intensity can only result in a constant shift of the mean of the variable distribution, but not the *shape *of the distribution. With this understanding in mind, we now proceed with the theory in a rather general setting.

Given a distribution function *g*(*x*) with ∫*g*(*x*)*dx *= 1, the *k*th moment is given by ⟨*x*^*k*^⟩ ≡ ∫*x*^*k*^*g*(*x*)*dx*. The first moment is the mean, and the difference between the second moment and first moment squared, ⟨*x*^2^⟩ – ⟨*x*⟩^2^, is the variance. The central limit theorem may be stated as follows [24]: If one samples independently *n *numbers, say *x*_1_, *x*_2_, ..., *x*_*n*_, from a given distribution function *g*(*x*) with mean x¯
 MathType@MTEF@5@5@+=feaafiart1ev1aaatCvAUfKttLearuWrP9MDH5MBPbIqV92AaeXatLxBI9gBaebbnrfifHhDYfgasaacH8akY=wiFfYdH8Gipec8Eeeu0xXdbba9frFj0=OqFfea0dXdd9vqai=hGuQ8kuc9pgc9s8qqaq=dirpe0xb9q8qiLsFr0=vr0=vr0dc8meaabaqaciaacaGaaeqabaqabeGadaaakeaacuWG4baEgaqeaaaa@2E3D@ and variance *σ*^2^, the distribution of the quantity y′≡n[(∑i=1nxi)/n−x¯]
 MathType@MTEF@5@5@+=feaafiart1ev1aaatCvAUfKttLearuWrP9MDH5MBPbIqV92AaeXatLxBI9gBaebbnrfifHhDYfgasaacH8akY=wiFfYdH8Gipec8Eeeu0xXdbba9frFj0=OqFfea0dXdd9vqai=hGuQ8kuc9pgc9s8qqaq=dirpe0xb9q8qiLsFr0=vr0=vr0dc8meaabaqaciaacaGaaeqabaqabeGadaaakeaacuWG5bqEgaqbaiabggMi6oaakaaabaGaemOBa4galeqaaOGaei4waSLaeiikaGYaaabmaeaacqWG4baEdaWgaaWcbaGaemyAaKgabeaaaeaacqWGPbqAcqGH9aqpcqaIXaqmaeaacqWGUbGBa0GaeyyeIuoakiabcMcaPiabc+caViabd6gaUjabgkHiTiqbdIha4zaaraGaeiyxa0faaa@4438@, a random number itself, will approach a Gaussian as *n *approaches infinity with zero mean and variance *σ*^2 ^provided that x¯
 MathType@MTEF@5@5@+=feaafiart1ev1aaatCvAUfKttLearuWrP9MDH5MBPbIqV92AaeXatLxBI9gBaebbnrfifHhDYfgasaacH8akY=wiFfYdH8Gipec8Eeeu0xXdbba9frFj0=OqFfea0dXdd9vqai=hGuQ8kuc9pgc9s8qqaq=dirpe0xb9q8qiLsFr0=vr0=vr0dc8meaabaqaciaacaGaaeqabaqabeGadaaakeaacuWG4baEgaqeaaaa@2E3D@ and *σ*^2 ^are finite. When dealing with finite *n*, one may simply consider y≡(∑i=1nxi)/n−x¯
 MathType@MTEF@5@5@+=feaafiart1ev1aaatCvAUfKttLearuWrP9MDH5MBPbIqV92AaeXatLxBI9gBaebbnrfifHhDYfgasaacH8akY=wiFfYdH8Gipec8Eeeu0xXdbba9frFj0=OqFfea0dXdd9vqai=hGuQ8kuc9pgc9s8qqaq=dirpe0xb9q8qiLsFr0=vr0=vr0dc8meaabaqaciaacaGaaeqabaqabeGadaaakeaacqWG5bqEcqGHHjIUcqGGOaakdaaeWaqaaiabdIha4naaBaaaleaacqWGPbqAaeqaaaqaaiabdMgaPjabg2da9iabigdaXaqaaiabd6gaUbqdcqGHris5aOGaeiykaKIaei4la8IaemOBa4MaeyOeI0IafmiEaGNbaebaaaa@4022@, and anticipate the distribution function of *y *to be *close *to Gaussian with zero mean and variance *σ*^2^/*n*.

The situation we wish to study is when *n *is not too large and when ⟨(*x *- x¯
 MathType@MTEF@5@5@+=feaafiart1ev1aaatCvAUfKttLearuWrP9MDH5MBPbIqV92AaeXatLxBI9gBaebbnrfifHhDYfgasaacH8akY=wiFfYdH8Gipec8Eeeu0xXdbba9frFj0=OqFfea0dXdd9vqai=hGuQ8kuc9pgc9s8qqaq=dirpe0xb9q8qiLsFr0=vr0=vr0dc8meaabaqaciaacaGaaeqabaqabeGadaaakeaacuWG4baEgaqeaaaa@2E3D@)^3^) ⟩ ≡ ∫(*x *- x¯
 MathType@MTEF@5@5@+=feaafiart1ev1aaatCvAUfKttLearuWrP9MDH5MBPbIqV92AaeXatLxBI9gBaebbnrfifHhDYfgasaacH8akY=wiFfYdH8Gipec8Eeeu0xXdbba9frFj0=OqFfea0dXdd9vqai=hGuQ8kuc9pgc9s8qqaq=dirpe0xb9q8qiLsFr0=vr0=vr0dc8meaabaqaciaacaGaaeqabaqabeGadaaakeaacuWG4baEgaqeaaaa@2E3D@)^3^)*g*(*x*)*dx *is not small, *i.e*., when the skewness of the distribution function is nonnegligible in the sense that the condition |⟨(*x *- x¯
 MathType@MTEF@5@5@+=feaafiart1ev1aaatCvAUfKttLearuWrP9MDH5MBPbIqV92AaeXatLxBI9gBaebbnrfifHhDYfgasaacH8akY=wiFfYdH8Gipec8Eeeu0xXdbba9frFj0=OqFfea0dXdd9vqai=hGuQ8kuc9pgc9s8qqaq=dirpe0xb9q8qiLsFr0=vr0=vr0dc8meaabaqaciaacaGaaeqabaqabeGadaaakeaacuWG4baEgaqeaaaa@2E3D@)^3 ^⟩| ≪ ∫ *n*⟨(*x *- x¯
 MathType@MTEF@5@5@+=feaafiart1ev1aaatCvAUfKttLearuWrP9MDH5MBPbIqV92AaeXatLxBI9gBaebbnrfifHhDYfgasaacH8akY=wiFfYdH8Gipec8Eeeu0xXdbba9frFj0=OqFfea0dXdd9vqai=hGuQ8kuc9pgc9s8qqaq=dirpe0xb9q8qiLsFr0=vr0=vr0dc8meaabaqaciaacaGaaeqabaqabeGadaaakeaacuWG4baEgaqeaaaa@2E3D@)^2^)⟩ is not true due to finite *n*. Inclusion of this term and other higher order odd moments introduces skewness to the distribution function of the *y *variable. The simplest choice, however, is to keep only the third moment. As for the higher order even moments, they contribute *symmetrically *to the probability distribution function of *y*, thus suppressing the skewness observed in the score distribution. We therefore choose to ignore all moments fourth or higher. To provide an analytical expression for the probability distribution function for *y *with nonnegligible skewness, we first show how the central limit theorem can be derived *heuristically *and how such a heuristic approach can be readily employed to give correct asymptotics that one needs. For simplicity, we will proceed under the assumption that x¯
 MathType@MTEF@5@5@+=feaafiart1ev1aaatCvAUfKttLearuWrP9MDH5MBPbIqV92AaeXatLxBI9gBaebbnrfifHhDYfgasaacH8akY=wiFfYdH8Gipec8Eeeu0xXdbba9frFj0=OqFfea0dXdd9vqai=hGuQ8kuc9pgc9s8qqaq=dirpe0xb9q8qiLsFr0=vr0=vr0dc8meaabaqaciaacaGaaeqabaqabeGadaaakeaacuWG4baEgaqeaaaa@2E3D@ ≡ ⟨*x*⟩ = ∫ *xg*(*x*)*dx *= 0. Extending the results obtained here to the case of nonzero but finite x¯
 MathType@MTEF@5@5@+=feaafiart1ev1aaatCvAUfKttLearuWrP9MDH5MBPbIqV92AaeXatLxBI9gBaebbnrfifHhDYfgasaacH8akY=wiFfYdH8Gipec8Eeeu0xXdbba9frFj0=OqFfea0dXdd9vqai=hGuQ8kuc9pgc9s8qqaq=dirpe0xb9q8qiLsFr0=vr0=vr0dc8meaabaqaciaacaGaaeqabaqabeGadaaakeaacuWG4baEgaqeaaaa@2E3D@ is straightforward. Note that by definition, we have *g*(*x*) ≥ 0 ∀ *x*. Also, we further assume that *g*(*x*) *> *0 over only a finite range of *x*, and define *X *≡ max{abs(*x*) *|g*(*x*) *> *0}.

By definition, we may write down the probability distribution function for *y *as

pdf(y)=∫⋯∫∏i=1n(g(xi)dxi)δ(y−1n∑i=1nxi),
 MathType@MTEF@5@5@+=feaafiart1ev1aaatCvAUfKttLearuWrP9MDH5MBPbIqV92AaeXatLxBI9gBaebbnrfifHhDYfgasaacH8akY=wiFfYdH8Gipec8Eeeu0xXdbba9frFj0=OqFfea0dXdd9vqai=hGuQ8kuc9pgc9s8qqaq=dirpe0xb9q8qiLsFr0=vr0=vr0dc8meaabaqaciaacaGaaeqabaqabeGadaaakeaafaqabeqacaaabaGaeeiCaaNaeeizaqMaeeOzayMaeiikaGIaemyEaKNaeiykaKIaeyypa0Zaa8qaaeaacqWIVlctdaWdbaqaamaarahabaGaeiikaGIaem4zaCMaeiikaGIaemiEaG3aaSbaaSqaaiabdMgaPbqabaGccqGGPaqkcqWGKbazcqWG4baEdaWgaaWcbaGaemyAaKgabeaakiabcMcaPaWcbaGaemyAaKMaeyypa0JaeGymaedabaGaemOBa4ganiabg+GivdaaleqabeqdcqGHRiI8aaWcbeqab0Gaey4kIipaaOqaaGGaciab=r7aKjabcIcaOiabdMha5jabgkHiTmaalaaabaGaeGymaedabaGaemOBa4gaamaaqahabaGaemiEaG3aaSbaaSqaaiabdMgaPbqabaaabaGaemyAaKMaeyypa0JaeGymaedabaGaemOBa4ganiabggHiLdGccqGGPaqkcqGGSaalaaaaaa@60EC@

where *δ *(*y – c*) is the Dirac delta function that has zero value everywhere except when *y *= *c *and has normalization ∫ *δ*(*y – c*)*dy *= 1. Upon introducing the integral representation of the Dirac delta function

δ(y−c)=12π∫−∞∞eik(y−c)dk,
 MathType@MTEF@5@5@+=feaafiart1ev1aaatCvAUfKttLearuWrP9MDH5MBPbIqV92AaeXatLxBI9gBaebbnrfifHhDYfgasaacH8akY=wiFfYdH8Gipec8Eeeu0xXdbba9frFj0=OqFfea0dXdd9vqai=hGuQ8kuc9pgc9s8qqaq=dirpe0xb9q8qiLsFr0=vr0=vr0dc8meaabaqaciaacaGaaeqabaqabeGadaaakeaaiiGacqWF0oazcqGGOaakcqWG5bqEcqGHsislcqWGJbWycqGGPaqkcqGH9aqpdaWcaaqaaiabigdaXaqaaiabikdaYiab=b8aWbaadaWdXaqaaiabdwgaLnaaCaaaleqabaGaemyAaKMaem4AaSMaeiikaGIaemyEaKNaeyOeI0Iaem4yamMaeiykaKcaaaqaaiabgkHiTiabg6HiLcqaaiabg6HiLcqdcqGHRiI8aOGaemizaqMaem4AaSMaeiilaWcaaa@4B9B@

we may rewrite pdf(*y*) as

pdf(y)=12π∫−∞∞eikydk[∫g(x)e−ikxndx]n=12π∫−∞∞exp⁡{iky+nln⁡[f(kn)]}dk
 MathType@MTEF@5@5@+=feaafiart1ev1aaatCvAUfKttLearuWrP9MDH5MBPbIqV92AaeXatLxBI9gBaebbnrfifHhDYfgasaacH8akY=wiFfYdH8Gipec8Eeeu0xXdbba9frFj0=OqFfea0dXdd9vqai=hGuQ8kuc9pgc9s8qqaq=dirpe0xb9q8qiLsFr0=vr0=vr0dc8meaabaqaciaacaGaaeqabaqabeGadaaakeaafaqaaeGadaaabaGaeeiCaaNaeeizaqMaeeOzayMaeiikaGIaemyEaKNaeiykaKcabaGaeyypa0dabaWaaSaaaeaacqaIXaqmaeaacqaIYaGmiiGacqWFapaCaaWaa8qmaeaacqWGLbqzdaahaaWcbeqaaiabdMgaPjabdUgaRjabdMha5baakiabdsgaKjabdUgaRbWcbaGaeyOeI0IaeyOhIukabaGaeyOhIukaniabgUIiYdGcdaWadaqaamaapeaabaGaem4zaCMaeiikaGIaemiEaGNaeiykaKIaemyzau2aaWbaaSqabeaacqGHsislcqWGPbqAdaWcaaqaaiabdUgaRjabdIha4bqaaiabd6gaUbaaaaGccqWGKbazcqWG4baEaSqabeqaniabgUIiYdaakiaawUfacaGLDbaadaahaaWcbeqaaiabd6gaUbaaaOqaaaqaaiabg2da9aqaamaalaaabaGaeGymaedabaGaeGOmaiJae8hWdahaamaapedabaGagiyzauMaeiiEaGNaeiiCaa3aaiWaaeaacqWGPbqAcqWGRbWAcqWG5bqEcqGHRaWkcqWGUbGBcyGGSbaBcqGGUbGBdaWadaqaaiabdAgaMnaabmaabaWaaSaaaeaacqWGRbWAaeaacqWGUbGBaaaacaGLOaGaayzkaaaacaGLBbGaayzxaaaacaGL7bGaayzFaaGaemizaqMaem4AaSgaleaacqGHsislcqGHEisPaeaacqGHEisPa0Gaey4kIipaaaaaaa@80C2@

where

f(kn)=∫g(x)e−iknxdx=∫g(x)[∑l=0∞(−i)ll!(kn)lxl]dx.
 MathType@MTEF@5@5@+=feaafiart1ev1aaatCvAUfKttLearuWrP9MDH5MBPbIqV92AaeXatLxBI9gBaebbnrfifHhDYfgasaacH8akY=wiFfYdH8Gipec8Eeeu0xXdbba9frFj0=OqFfea0dXdd9vqai=hGuQ8kuc9pgc9s8qqaq=dirpe0xb9q8qiLsFr0=vr0=vr0dc8meaabaqaciaacaGaaeqabaqabeGadaaakeaafaqaaeGadaaabaGaemOzay2aaeWaaeaadaWcaaqaaiabdUgaRbqaaiabd6gaUbaaaiaawIcacaGLPaaaaeaacqGH9aqpaeaadaWdbaqaaiabdEgaNjabcIcaOiabdIha4jabcMcaPiabdwgaLnaaCaaaleqabaGaeyOeI0IaemyAaK2aaSaaaeaacqWGRbWAaeaacqWGUbGBaaGaemiEaGhaaOGaemizaqMaemiEaGhaleqabeqdcqGHRiI8aaGcbaaabaGaeyypa0dabaWaa8qaaeaacqWGNbWzcqGGOaakcqWG4baEcqGGPaqkaSqabeqaniabgUIiYdGcdaWadaqaamaaqahabaWaaSaaaeaacqGGOaakcqGHsislcqWGPbqAcqGGPaqkdaahaaWcbeqaaiabdYgaSbaaaOqaaiabdYgaSjabcgcaHaaaaSqaaiabdYgaSjabg2da9iabicdaWaqaaiabg6HiLcqdcqGHris5aOWaaeWaaeaadaWcaaqaaiabdUgaRbqaaiabd6gaUbaaaiaawIcacaGLPaaadaahaaWcbeqaaiabdYgaSbaakiabdIha4naaCaaaleqabaGaemiBaWgaaaGccaGLBbGaayzxaaGaemizaqMaemiEaGNaeiOla4caaaaa@69F8@

Since we assume that *g*(*x*) *> *0 only over a finite range of *x*, all moments ⟨*x*^*l *^⟩ have their absolute values |⟨*x*^*l*^⟩| bounded by *X*^*l*^. This implies that we may exchange the order of the sum and the integral in Eq. (5) and arrive at

f(kn)=∑l=0∞(−i)ll!(kn)l〈xl〉.
 MathType@MTEF@5@5@+=feaafiart1ev1aaatCvAUfKttLearuWrP9MDH5MBPbIqV92AaeXatLxBI9gBaebbnrfifHhDYfgasaacH8akY=wiFfYdH8Gipec8Eeeu0xXdbba9frFj0=OqFfea0dXdd9vqai=hGuQ8kuc9pgc9s8qqaq=dirpe0xb9q8qiLsFr0=vr0=vr0dc8meaabaqaciaacaGaaeqabaqabeGadaaakeaacqWGMbGzdaqadaqaamaalaaabaGaem4AaSgabaGaemOBa4gaaaGaayjkaiaawMcaaiabg2da9maaqahabaWaaSaaaeaacqGGOaakcqGHsislcqWGPbqAcqGGPaqkdaahaaWcbeqaaiabdYgaSbaaaOqaaiabdYgaSjabcgcaHaaaaSqaaiabdYgaSjabg2da9iabicdaWaqaaiabg6HiLcqdcqGHris5aOWaaeWaaeaadaWcaaqaaiabdUgaRbqaaiabd6gaUbaaaiaawIcacaGLPaaadaahaaWcbeqaaiabdYgaSbaakiabgMYiHlabdIha4naaCaaaleqabaGaemiBaWgaaOGaeyOkJeVaeiOla4caaa@4FB0@

In the limit of large *n*, one may keep only the first few terms in Eq. (6). In particular, if one only keeps up to the *l *= 2 term, we have

f(kn)=1−12(kn)2〈x2〉+O(n−3),
 MathType@MTEF@5@5@+=feaafiart1ev1aaatCvAUfKttLearuWrP9MDH5MBPbIqV92AaeXatLxBI9gBaebbnrfifHhDYfgasaacH8akY=wiFfYdH8Gipec8Eeeu0xXdbba9frFj0=OqFfea0dXdd9vqai=hGuQ8kuc9pgc9s8qqaq=dirpe0xb9q8qiLsFr0=vr0=vr0dc8meaabaqaciaacaGaaeqabaqabeGadaaakeaacqWGMbGzdaqadaqaamaalaaabaGaem4AaSgabaGaemOBa4gaaaGaayjkaiaawMcaaiabg2da9iabigdaXiabgkHiTmaalaaabaGaeGymaedabaGaeGOmaidaamaabmaabaWaaSaaaeaacqWGRbWAaeaacqWGUbGBaaaacaGLOaGaayzkaaWaaWbaaSqabeaacqaIYaGmaaGccqGHPms4cqWG4baEdaahaaWcbeqaaiabikdaYaaakiabgQYiXlabgUcaRmrtHrhAL1wy0L2yHvtyaeHbnfgDOvwBHrxAJfwnaGabaiab=5q8pjabcIcaOiabd6gaUnaaCaaaleqabaGaeyOeI0IaeG4mamdaaOGaeiykaKIaeiilaWcaaa@555F@

and consequently

nln⁡[f(kn)]=−k22n〈x2〉+O(n−2).
 MathType@MTEF@5@5@+=feaafiart1ev1aaatCvAUfKttLearuWrP9MDH5MBPbIqV92AaeXatLxBI9gBaebbnrfifHhDYfgasaacH8akY=wiFfYdH8Gipec8Eeeu0xXdbba9frFj0=OqFfea0dXdd9vqai=hGuQ8kuc9pgc9s8qqaq=dirpe0xb9q8qiLsFr0=vr0=vr0dc8meaabaqaciaacaGaaeqabaqabeGadaaakeaacqWGUbGBcyGGSbaBcqGGUbGBdaWadaqaaiabdAgaMnaabmaabaWaaSaaaeaacqWGRbWAaeaacqWGUbGBaaaacaGLOaGaayzkaaaacaGLBbGaayzxaaGaeyypa0JaeyOeI0YaaSaaaeaacqWGRbWAdaahaaWcbeqaaiabikdaYaaaaOqaaiabikdaYiabd6gaUbaacqGHPms4cqWG4baEdaahaaWcbeqaaiabikdaYaaakiabgQYiXlabgUcaRmrtHrhAL1wy0L2yHvtyaeHbnfgDOvwBHrxAJfwnaGabaiab=5q8pjabcIcaOiabd6gaUnaaCaaaleqabaGaeyOeI0IaeGOmaidaaOGaeiykaKIaeiOla4caaa@5805@

Therefore

pdf(f)=12π∫−∞∞exp⁡{iky−k22n〈x2〉+O(n−2)}dx≈12π〈x2〉/nexp⁡[−y22〈x2〉/n],
 MathType@MTEF@5@5@+=feaafiart1ev1aaatCvAUfKttLearuWrP9MDH5MBPbIqV92AaeXatLxBI9gBaebbnrfifHhDYfgasaacH8akY=wiFfYdH8Gipec8Eeeu0xXdbba9frFj0=OqFfea0dXdd9vqai=hGuQ8kuc9pgc9s8qqaq=dirpe0xb9q8qiLsFr0=vr0=vr0dc8meaabaqaciaacaGaaeqabaqabeGadaaakeaafaqaaeGadaaabaGaeeiCaaNaeeizaqMaeeOzayMaeiikaGIaemOzayMaeiykaKcabaGaeyypa0dabaWaaSaaaeaacqaIXaqmaeaacqaIYaGmiiGacqWFapaCaaWaa8qmaeaacyGGLbqzcqGG4baEcqGGWbaCdaGadaqaaiabdMgaPjabdUgaRjabdMha5jabgkHiTmaalaaabaGaem4AaS2aaWbaaSqabeaacqaIYaGmaaaakeaacqaIYaGmcqWGUbGBaaGaeyykJeUaemiEaG3aaWbaaSqabeaacqaIYaGmaaGccqGHQms8cqGHRaWkt0uy0HwzTfgDPnwy1egaryqtHrhAL1wy0L2yHvdaiqaacqGFoe=tcqGGOaakcqWGUbGBdaahaaWcbeqaaiabgkHiTiabikdaYaaakiabcMcaPaGaay5Eaiaaw2haaaWcbaGaeyOeI0IaeyOhIukabaGaeyOhIukaniabgUIiYdGccqWGKbazcqWG4baEaeaaaeaacqGHijYUaeaadaWcaaqaaiabigdaXaqaamaakaaabaGaeGOmaiJae8hWdaNaeyykJeUaemiEaG3aaWbaaSqabeaacqaIYaGmaaGccqGHQms8cqGGVaWlcqWGUbGBaSqabaaaaOGagiyzauMaeiiEaGNaeiiCaa3aamWaaeaacqGHsisldaWcaaqaaiabdMha5naaCaaaleqabaGaeGOmaidaaaGcbaGaeGOmaiJaeyykJeUaemiEaG3aaWbaaSqabeaacqaIYaGmaaGccqGHQms8cqGGVaWlcqWGUbGBaaaacaGLBbGaayzxaaGaeiilaWcaaaaa@8B88@

which is the celebrated Gaussian distribution of the central limit theorem.

In this section, we investigate the consequence of non-negligible skewness. Specifically, in the expansion of Eq. (5), what happens if |*k*^3^⟨*x*^3^⟩/*n*^3^| ≪ |*k*^2^⟨*x*^2^⟩/*n*^2^| is not true while all other higher moments become negligible. In this case, we will have to keep one more term in f(kn)
 MathType@MTEF@5@5@+=feaafiart1ev1aaatCvAUfKttLearuWrP9MDH5MBPbIqV92AaeXatLxBI9gBaebbnrfifHhDYfgasaacH8akY=wiFfYdH8Gipec8Eeeu0xXdbba9frFj0=OqFfea0dXdd9vqai=hGuQ8kuc9pgc9s8qqaq=dirpe0xb9q8qiLsFr0=vr0=vr0dc8meaabaqaciaacaGaaeqabaqabeGadaaakeaacqWGMbGzdaqadaqaamaalaaabaGaem4AaSgabaGaemOBa4gaaaGaayjkaiaawMcaaaaa@325E@ than is done in Eq. (7) and arrive at

f(kn)=1−k22n2〈x2〉+ik36n3〈x3〉+O(n−4).
 MathType@MTEF@5@5@+=feaafiart1ev1aaatCvAUfKttLearuWrP9MDH5MBPbIqV92AaeXatLxBI9gBaebbnrfifHhDYfgasaacH8akY=wiFfYdH8Gipec8Eeeu0xXdbba9frFj0=OqFfea0dXdd9vqai=hGuQ8kuc9pgc9s8qqaq=dirpe0xb9q8qiLsFr0=vr0=vr0dc8meaabaqaciaacaGaaeqabaqabeGadaaakeaacqWGMbGzdaqadaqaamaalaaabaGaem4AaSgabaGaemOBa4gaaaGaayjkaiaawMcaaiabg2da9iabigdaXiabgkHiTmaalaaabaGaem4AaS2aaWbaaSqabeaacqaIYaGmaaaakeaacqaIYaGmcqWGUbGBdaahaaWcbeqaaiabikdaYaaaaaGccqGHPms4cqWG4baEdaahaaWcbeqaaiabikdaYaaakiabgQYiXlabgUcaRmaalaaabaGaemyAaKMaem4AaS2aaWbaaSqabeaacqaIZaWmaaaakeaacqaI2aGncqWGUbGBdaahaaWcbeqaaiabiodaZaaaaaGccqGHPms4cqWG4baEdaahaaWcbeqaaiabiodaZaaakiabgQYiXlabgUcaRmrtHrhAL1wy0L2yHvtyaeHbnfgDOvwBHrxAJfwnaGabaiab=5q8pjabcIcaOiabd6gaUnaaCaaaleqabaGaeyOeI0IaeGinaqdaaOGaeiykaKIaeiOla4caaa@628D@

This then leads to

nln⁡[f(kn)]=−k22n〈x2〉+ik36n2〈x3〉+O(n−3).
 MathType@MTEF@5@5@+=feaafiart1ev1aaatCvAUfKttLearuWrP9MDH5MBPbIqV92AaeXatLxBI9gBaebbnrfifHhDYfgasaacH8akY=wiFfYdH8Gipec8Eeeu0xXdbba9frFj0=OqFfea0dXdd9vqai=hGuQ8kuc9pgc9s8qqaq=dirpe0xb9q8qiLsFr0=vr0=vr0dc8meaabaqaciaacaGaaeqabaqabeGadaaakeaacqWGUbGBcyGGSbaBcqGGUbGBdaWadaqaaiabdAgaMnaabmaabaWaaSaaaeaacqWGRbWAaeaacqWGUbGBaaaacaGLOaGaayzkaaaacaGLBbGaayzxaaGaeyypa0JaeyOeI0YaaSaaaeaacqWGRbWAdaahaaWcbeqaaiabikdaYaaaaOqaaiabikdaYiabd6gaUbaacqGHPms4cqWG4baEdaahaaWcbeqaaiabikdaYaaakiabgQYiXlabgUcaRiabdMgaPnaalaaabaGaem4AaS2aaWbaaSqabeaacqaIZaWmaaaakeaacqaI2aGncqWGUbGBdaahaaWcbeqaaiabikdaYaaaaaGccqGHPms4cqWG4baEdaahaaWcbeqaaiabiodaZaaakiabgQYiXlabgUcaRmrtHrhAL1wy0L2yHvtyaeHbnfgDOvwBHrxAJfwnaGabaiab=5q8pjabcIcaOiabd6gaUnaaCaaaleqabaGaeyOeI0IaeG4mamdaaOGaeiykaKIaeiOla4caaa@668D@

Using the definition of pdf(*y*) in Eq. (4), we now have

pdf(y)≈12π∫−∞∞exp⁡{iky−k22n〈x2〉+ik36n2〈x3〉}dk.
 MathType@MTEF@5@5@+=feaafiart1ev1aaatCvAUfKttLearuWrP9MDH5MBPbIqV92AaeXatLxBI9gBaebbnrfifHhDYfgasaacH8akY=wiFfYdH8Gipec8Eeeu0xXdbba9frFj0=OqFfea0dXdd9vqai=hGuQ8kuc9pgc9s8qqaq=dirpe0xb9q8qiLsFr0=vr0=vr0dc8meaabaqaciaacaGaaeqabaqabeGadaaakeaacqqGWbaCcqqGKbazcqqGMbGzcqGGOaakcqWG5bqEcqGGPaqkcqGHijYUdaWcaaqaaiabigdaXaqaaiabikdaYGGaciab=b8aWbaadaWdXaqaaiGbcwgaLjabcIha4jabcchaWnaacmaabaGaemyAaKMaem4AaSMaemyEaKNaeyOeI0YaaSaaaeaacqWGRbWAdaahaaWcbeqaaiabikdaYaaaaOqaaiabikdaYiabd6gaUbaacqGHPms4cqWG4baEdaahaaWcbeqaaiabikdaYaaakiabgQYiXlabgUcaRiabdMgaPnaalaaabaGaem4AaS2aaWbaaSqabeaacqaIZaWmaaaakeaacqaI2aGncqWGUbGBdaahaaWcbeqaaiabikdaYaaaaaGccqGHPms4cqWG4baEdaahaaWcbeqaaiabiodaZaaakiabgQYiXdGaay5Eaiaaw2haaaWcbaGaeyOeI0IaeyOhIukabaGaeyOhIukaniabgUIiYdGccqWGKbazcqWGRbWAcqGGUaGlaaa@6800@

Employing the saddle-point approximation, we seek the complex valued k* such that

∂∂k[iky−k22n〈x2〉+ik36n2〈k3〉]k=k∗=0.
 MathType@MTEF@5@5@+=feaafiart1ev1aaatCvAUfKttLearuWrP9MDH5MBPbIqV92AaeXatLxBI9gBaebbnrfifHhDYfgasaacH8akY=wiFfYdH8Gipec8Eeeu0xXdbba9frFj0=OqFfea0dXdd9vqai=hGuQ8kuc9pgc9s8qqaq=dirpe0xb9q8qiLsFr0=vr0=vr0dc8meaabaqaciaacaGaaeqabaqabeGadaaakeaadaWcaaqaaiabgkGi2cqaaiabgkGi2kabdUgaRbaadaWadaqaaiabdMgaPjabdUgaRjabdMha5jabgkHiTmaalaaabaGaem4AaS2aaWbaaSqabeaacqaIYaGmaaaakeaacqaIYaGmcqWGUbGBaaGaeyykJeUaemiEaG3aaWbaaSqabeaacqaIYaGmaaGccqGHQms8cqGHRaWkcqWGPbqAdaWcaaqaaiabdUgaRnaaCaaaleqabaGaeG4mamdaaaGcbaGaeGOnayJaemOBa42aaWbaaSqabeaacqaIYaGmaaaaaOGaeyykJeUaem4AaS2aaWbaaSqabeaacqaIZaWmaaGccqGHQms8aiaawUfacaGLDbaadaWgaaWcbaGaem4AaSMaeyypa0Jaem4AaS2aaWbaaWqabeaacqGHxiIkaaaaleqaaOGaeyypa0JaeGimaaJaeiOla4caaa@5975@

A quadratic equation for k* is obtained

iy−〈x2〉nk∗+i〈x3〉2n2k∗2=0.
 MathType@MTEF@5@5@+=feaafiart1ev1aaatCvAUfKttLearuWrP9MDH5MBPbIqV92AaeXatLxBI9gBaebbnrfifHhDYfgasaacH8akY=wiFfYdH8Gipec8Eeeu0xXdbba9frFj0=OqFfea0dXdd9vqai=hGuQ8kuc9pgc9s8qqaq=dirpe0xb9q8qiLsFr0=vr0=vr0dc8meaabaqaciaacaGaaeqabaqabeGadaaakeaacqWGPbqAcqWG5bqEcqGHsisldaWcaaqaaiabgMYiHlabdIha4naaCaaaleqabaGaeGOmaidaaOGaeyOkJepabaGaemOBa4gaaiabdUgaRnaaCaaaleqabaGaey4fIOcaaOGaey4kaSIaemyAaK2aaSaaaeaacqGHPms4cqWG4baEdaahaaWcbeqaaiabiodaZaaakiabgQYiXdqaaiabikdaYiabd6gaUnaaCaaaleqabaGaeGOmaidaaaaakiabdUgaRnaaCaaaleqabaGaey4fIOIaeGOmaidaaOGaeyypa0JaeGimaaJaeiOla4caaa@4CD1@

The two solutions are given by

k∗=n〈x2〉1β[−i±−1−2βy]
 MathType@MTEF@5@5@+=feaafiart1ev1aaatCvAUfKttLearuWrP9MDH5MBPbIqV92AaeXatLxBI9gBaebbnrfifHhDYfgasaacH8akY=wiFfYdH8Gipec8Eeeu0xXdbba9frFj0=OqFfea0dXdd9vqai=hGuQ8kuc9pgc9s8qqaq=dirpe0xb9q8qiLsFr0=vr0=vr0dc8meaabaqaciaacaGaaeqabaqabeGadaaakeaacqWGRbWAdaahaaWcbeqaaiabgEHiQaaakiabg2da9maalaaabaGaemOBa4gabaGaeyykJeUaemiEaG3aaWbaaSqabeaacqaIYaGmaaGccqGHQms8aaWaaSaaaeaacqaIXaqmaeaaiiGacqWFYoGyaaWaamWaaeaacqGHsislcqWGPbqAcqGHXcqSdaGcaaqaaiabgkHiTiabigdaXiabgkHiTiabikdaYiab=j7aIjabdMha5bWcbeaaaOGaay5waiaaw2faaaaa@4799@

where *β *= ⟨*x*⟩^3^/⟨*x*^2^⟩^2^. In general, it is possible for *β *(or ⟨*x*⟩ ^3^) to be positive or negative. For the purpose of scoring MS^2 ^spectra, however, we are dealing with the case where *β *> 0. The treatment when 1 + 2*βy *< 0 is exactly parallel to what will be done next, and is therefore omitted. Consequently, we have

k∗=−in〈x2〉1β[1±1+2βy].
 MathType@MTEF@5@5@+=feaafiart1ev1aaatCvAUfKttLearuWrP9MDH5MBPbIqV92AaeXatLxBI9gBaebbnrfifHhDYfgasaacH8akY=wiFfYdH8Gipec8Eeeu0xXdbba9frFj0=OqFfea0dXdd9vqai=hGuQ8kuc9pgc9s8qqaq=dirpe0xb9q8qiLsFr0=vr0=vr0dc8meaabaqaciaacaGaaeqabaqabeGadaaakeaacqWGRbWAdaahaaWcbeqaaiabgEHiQaaakiabg2da9iabgkHiTiabdMgaPnaalaaabaGaemOBa4gabaGaeyykJeUaemiEaG3aaWbaaSqabeaacqaIYaGmaaGccqGHQms8aaWaaSaaaeaacqaIXaqmaeaaiiGacqWFYoGyaaWaamWaaeaacqaIXaqmcqGHXcqSdaGcaaqaaiabigdaXiabgUcaRiabikdaYiab=j7aIjabdMha5bWcbeaaaOGaay5waiaaw2faaiabc6caUaaa@4875@

Note that in the limit of negligible skewness (*β *→ 0), one should recover the Gaussian case which corresponds to

k∗=in〈x2〉y.
 MathType@MTEF@5@5@+=feaafiart1ev1aaatCvAUfKttLearuWrP9MDH5MBPbIqV92AaeXatLxBI9gBaebbnrfifHhDYfgasaacH8akY=wiFfYdH8Gipec8Eeeu0xXdbba9frFj0=OqFfea0dXdd9vqai=hGuQ8kuc9pgc9s8qqaq=dirpe0xb9q8qiLsFr0=vr0=vr0dc8meaabaqaciaacaGaaeqabaqabeGadaaakeaacqWGRbWAdaahaaWcbeqaaiabgEHiQaaakiabg2da9iabdMgaPnaalaaabaGaemOBa4gabaGaeyykJeUaemiEaG3aaWbaaSqabeaacqaIYaGmaaGccqGHQms8aaGaemyEaKNaeiOla4caaa@3B8B@

Therefore, we must take the solution that has the right limit as *β *→ 0. This naturally leads to the choice

k∗=in〈x2〉1β[1+2βy−1].
 MathType@MTEF@5@5@+=feaafiart1ev1aaatCvAUfKttLearuWrP9MDH5MBPbIqV92AaeXatLxBI9gBaebbnrfifHhDYfgasaacH8akY=wiFfYdH8Gipec8Eeeu0xXdbba9frFj0=OqFfea0dXdd9vqai=hGuQ8kuc9pgc9s8qqaq=dirpe0xb9q8qiLsFr0=vr0=vr0dc8meaabaqaciaacaGaaeqabaqabeGadaaakeaacqWGRbWAdaahaaWcbeqaaiabgEHiQaaakiabg2da9iabdMgaPnaalaaabaGaemOBa4gabaGaeyykJeUaemiEaG3aaWbaaSqabeaacqaIYaGmaaGccqGHQms8aaWaaSaaaeaacqaIXaqmaeaaiiGacqWFYoGyaaWaamWaaeaadaGcaaqaaiabigdaXiabgUcaRiabikdaYiab=j7aIjabdMha5bWcbeaakiabgkHiTiabigdaXaGaay5waiaaw2faaiabc6caUaaa@4687@

One should then expand the exponent of the integrand in Eq. (12) in terms of (k – k*). After some algebra, one may rewrite the exponent of interest as

ik∗y−k∗22n〈x2〉+ik∗36n2〈x3〉−〈x2〉2n(k−k∗)2+i〈x3〉6n2[3k∗(k−k∗)2+(k−k∗)3]=n6〈x2〉β2[1−1+2βy][1+4βy−1+2βy]−〈x2〉2n1+2βy(k−k∗)2+i〈x3〉6n2(k−k∗)3.
 MathType@MTEF@5@5@+=feaafiart1ev1aaatCvAUfKttLearuWrP9MDH5MBPbIqV92AaeXatLxBI9gBaebbnrfifHhDYfgasaacH8akY=wiFfYdH8Gipec8Eeeu0xXdbba9frFj0=OqFfea0dXdd9vqai=hGuQ8kuc9pgc9s8qqaq=dirpe0xb9q8qiLsFr0=vr0=vr0dc8meaabaqaciaacaGaaeqabaqabeGadaaakeaafaqaaeabbaaaaeaacqWGPbqAcqWGRbWAdaahaaWcbeqaaiabgEHiQaaakiabdMha5jabgkHiTmaalaaabaGaem4AaS2aaWbaaSqabeaacqGHxiIkcqaIYaGmaaaakeaacqaIYaGmcqWGUbGBaaGaeyykJeUaemiEaG3aaWbaaSqabeaacqaIYaGmaaGccqGHQms8cqGHRaWkcqWGPbqAdaWcaaqaaiabdUgaRnaaCaaaleqabaGaey4fIOIaeG4mamdaaaGcbaGaeGOnayJaemOBa42aaWbaaSqabeaacqaIYaGmaaaaaOGaeyykJeUaemiEaG3aaWbaaSqabeaacqaIZaWmaaGccqGHQms8aeGabaanciaaxMaacqGHsisldaWcaaqaaiabgMYiHlabdIha4naaCaaaleqabaGaeGOmaidaaOGaeyOkJepabaGaeGOmaiJaemOBa4gaaiabcIcaOiabdUgaRjabgkHiTiabdUgaRnaaCaaaleqabaGaey4fIOcaaOGaeiykaKYaaWbaaSqabeaacqaIYaGmaaGccqGHRaWkcqWGPbqAdaWcaaqaaiabgMYiHlabdIha4naaCaaaleqabaGaeG4mamdaaOGaeyOkJepabaGaeGOnayJaemOBa42aaWbaaSqabeaacqaIYaGmaaaaaOWaamWaaeaacqaIZaWmcqWGRbWAdaahaaWcbeqaaiabgEHiQaaakiabcIcaOiabdUgaRjabgkHiTiabdUgaRnaaCaaaleqabaGaey4fIOcaaOGaeiykaKYaaWbaaSqabeaacqaIYaGmaaGccqGHRaWkcqGGOaakcqWGRbWAcqGHsislcqWGRbWAdaahaaWcbeqaaiabgEHiQaaakiabcMcaPmaaCaaaleqabaGaeG4mamdaaaGccaGLBbGaayzxaaaabiqaaaBacaWLjaGaeyypa0ZaaSaaaeaacqWGUbGBaeaacqaI2aGncqGHPms4cqWG4baEdaahaaWcbeqaaiabikdaYaaakiabgQYiXJGaciab=j7aInaaCaaaleqabaGaeGOmaidaaaaakmaadmaabaGaeGymaeJaeyOeI0YaaOaaaeaacqaIXaqmcqGHRaWkcqaIYaGmcqWFYoGycqWG5bqEaSqabaaakiaawUfacaGLDbaadaWadaqaaiabigdaXiabgUcaRiabisda0iab=j7aIjabdMha5jabgkHiTmaakaaabaGaeGymaeJaey4kaSIaeGOmaiJae8NSdiMaemyEaKhaleqaaaGccaGLBbGaayzxaaaabiqaaqJacaWLjaGaeyOeI0YaaSaaaeaacqGHPms4cqWG4baEdaahaaWcbeqaaiabikdaYaaakiabgQYiXdqaaiabikdaYiabd6gaUbaadaGcaaqaaiabigdaXiabgUcaRiabikdaYiab=j7aIjabdMha5bWcbeaakiabcIcaOiabdUgaRjabgkHiTiabdUgaRnaaCaaaleqabaGaey4fIOcaaOGaeiykaKYaaWbaaSqabeaacqaIYaGmaaGccqGHRaWkcqWGPbqAdaWcaaqaaiabgMYiHlabdIha4naaCaaaleqabaGaeG4mamdaaOGaeyOkJepabaGaeGOnayJaemOBa42aaWbaaSqabeaacqaIYaGmaaaaaOGaeiikaGIaem4AaSMaeyOeI0Iaem4AaS2aaWbaaSqabeaacqGHxiIkaaGccqGGPaqkdaahaaWcbeqaaiabiodaZaaakiabc6caUaaaaaa@D4FB@

One then obtains

pdf(y)≈Cexp⁡{nβ−26〈x2〉[1−1+2βy][1+4βy−1+2βy]}
 MathType@MTEF@5@5@+=feaafiart1ev1aaatCvAUfKttLearuWrP9MDH5MBPbIqV92AaeXatLxBI9gBaebbnrfifHhDYfgasaacH8akY=wiFfYdH8Gipec8Eeeu0xXdbba9frFj0=OqFfea0dXdd9vqai=hGuQ8kuc9pgc9s8qqaq=dirpe0xb9q8qiLsFr0=vr0=vr0dc8meaabaqaciaacaGaaeqabaqabeGadaaakeaafaqaaeGabaaabaGaeeiCaaNaeeizaqMaeeOzayMaeiikaGIaemyEaKNaeiykaKIaeyisISlabaWenfgDOvwBHrxAJfwnHbqeg0uy0HwzTfgDPnwy1aaceaGae8NaXpKagiyzauMaeiiEaGNaeiiCaa3aaiWaaeaadaWcaaqaaiabd6gaUHGaciab+j7aInaaCaaaleqabaGaeyOeI0IaeGOmaidaaaGcbaGaeGOnayJaeyykJeUaemiEaG3aaWbaaSqabeaacqaIYaGmaaGccqGHQms8aaWaamWaaeaacqaIXaqmcqGHsisldaGcaaqaaiabigdaXiabgUcaRiabikdaYiab+j7aIjabdMha5bWcbeaaaOGaay5waiaaw2faamaadmaabaGaeGymaeJaey4kaSIaeGinaqJae4NSdiMaemyEaKNaeyOeI0YaaOaaaeaacqaIXaqmcqGHRaWkcqaIYaGmcqGFYoGycqWG5bqEaSqabaaakiaawUfacaGLDbaaaiaawUhacaGL9baaaaaaaa@6C57@

where the constant C
 MathType@MTEF@5@5@+=feaafiart1ev1aaatCvAUfKttLearuWrP9MDH5MBPbIqV92AaeXatLxBI9gBaebbnrfifHhDYfgasaacH8akY=wiFfYdH8Gipec8Eeeu0xXdbba9frFj0=OqFfea0dXdd9vqai=hGuQ8kuc9pgc9s8qqaq=dirpe0xb9q8qiLsFr0=vr0=vr0dc8meaabaqaciaacaGaaeqabaqabeGadaaakeaat0uy0HwzTfgDPnwy1egaryqtHrhAL1wy0L2yHvdaiqaacqWFce=qaaa@3824@ actually has weak *y*-dependence and can be formally written as

C=12π∫e−〈x2〉2n1+2βy(k−k∗)2+i〈x3〉6n2(k−k∗)3+O(k4/n3)dk.
 MathType@MTEF@5@5@+=feaafiart1ev1aaatCvAUfKttLearuWrP9MDH5MBPbIqV92AaeXatLxBI9gBaebbnrfifHhDYfgasaacH8akY=wiFfYdH8Gipec8Eeeu0xXdbba9frFj0=OqFfea0dXdd9vqai=hGuQ8kuc9pgc9s8qqaq=dirpe0xb9q8qiLsFr0=vr0=vr0dc8meaabaqaciaacaGaaeqabaqabeGadaaakeaat0uy0HwzTfgDPnwy1egaryqtHrhAL1wy0L2yHvdaiqaacqWFce=qcqGH9aqpdaWcaaqaaiabigdaXaqaaiabikdaYGGaciab+b8aWbaadaWdbaqaaiabdwgaLnaaCaaaleqabaGaeyOeI0YaaSaaaeaacqGHPms4cqWG4baEdaahaaadbeqaaiabikdaYaaaliabgQYiXdqaaiabikdaYiabd6gaUbaadaGcaaqaaiabigdaXiabgUcaRiabikdaYiab+j7aIjabdMha5badbeaaliabcIcaOiabdUgaRjabgkHiTiabdUgaRnaaCaaameqabaGaey4fIOcaaSGaeiykaKYaaWbaaWqabeaacqaIYaGmaaWccqGHRaWkcqWGPbqAdaWcaaqaaiabgMYiHlabdIha4naaCaaameqabaGaeG4mamdaaSGaeyOkJepabaGaeGOnayJaemOBa42aaWbaaWqabeaacqaIYaGmaaaaaSGaeiikaGIaem4AaSMaeyOeI0Iaem4AaS2aaWbaaWqabeaacqGHxiIkaaWccqGGPaqkdaahaaadbeqaaiabiodaZaaaliabgUcaRiab=5q8pjabcIcaOiabdUgaRnaaCaaameqabaGaeGinaqdaaSGaei4la8IaemOBa42aaWbaaWqabeaacqaIZaWmaaWccqGGPaqkaaGccqWGKbazcqWGRbWAaSqabeqaniabgUIiYdGccqGGUaGlaaa@794D@

In principle, one may choose to retain the Gaussian part and expand the rest of the exponents in powers of (*k *– k*). We did not pursue this route, however, since such expansion will require information about higher cumulants of *g*(*x*) and experimentally *g*(*x*) may already contain some uncertainties. Instead, we simply treat C
 MathType@MTEF@5@5@+=feaafiart1ev1aaatCvAUfKttLearuWrP9MDH5MBPbIqV92AaeXatLxBI9gBaebbnrfifHhDYfgasaacH8akY=wiFfYdH8Gipec8Eeeu0xXdbba9frFj0=OqFfea0dXdd9vqai=hGuQ8kuc9pgc9s8qqaq=dirpe0xb9q8qiLsFr0=vr0=vr0dc8meaabaqaciaacaGaaeqabaqabeGadaaakeaat0uy0HwzTfgDPnwy1egaryqtHrhAL1wy0L2yHvdaiqaacqWFce=qaaa@3824@ as an integration constant.

Note that in our asymptotic expansion, 1 + 2*βy *> 0 is required, and therefore the derived expression loses its validity when 1 + 2*βy *→ 0^+^. Nonetheless, the result is valid for the *y *≫ 1 tail that one will be particularly interested in while assigning statistical significance. As for the determination of C
 MathType@MTEF@5@5@+=feaafiart1ev1aaatCvAUfKttLearuWrP9MDH5MBPbIqV92AaeXatLxBI9gBaebbnrfifHhDYfgasaacH8akY=wiFfYdH8Gipec8Eeeu0xXdbba9frFj0=OqFfea0dXdd9vqai=hGuQ8kuc9pgc9s8qqaq=dirpe0xb9q8qiLsFr0=vr0=vr0dc8meaabaqaciaacaGaaeqabaqabeGadaaakeaat0uy0HwzTfgDPnwy1egaryqtHrhAL1wy0L2yHvdaiqaacqWFce=qaaa@3824@, one may plot both the theoretical pdf (from Eq. (17) without C
 MathType@MTEF@5@5@+=feaafiart1ev1aaatCvAUfKttLearuWrP9MDH5MBPbIqV92AaeXatLxBI9gBaebbnrfifHhDYfgasaacH8akY=wiFfYdH8Gipec8Eeeu0xXdbba9frFj0=OqFfea0dXdd9vqai=hGuQ8kuc9pgc9s8qqaq=dirpe0xb9q8qiLsFr0=vr0=vr0dc8meaabaqaciaacaGaaeqabaqabeGadaaakeaat0uy0HwzTfgDPnwy1egaryqtHrhAL1wy0L2yHvdaiqaacqWFce=qaaa@3824@) and the experimentally obtained pdf on a linear-log plot. The amount of vertical displacement should give us ln C
 MathType@MTEF@5@5@+=feaafiart1ev1aaatCvAUfKttLearuWrP9MDH5MBPbIqV92AaeXatLxBI9gBaebbnrfifHhDYfgasaacH8akY=wiFfYdH8Gipec8Eeeu0xXdbba9frFj0=OqFfea0dXdd9vqai=hGuQ8kuc9pgc9s8qqaq=dirpe0xb9q8qiLsFr0=vr0=vr0dc8meaabaqaciaacaGaaeqabaqabeGadaaakeaat0uy0HwzTfgDPnwy1egaryqtHrhAL1wy0L2yHvdaiqaacqWFce=qaaa@3824@ and can be used to obtain C
 MathType@MTEF@5@5@+=feaafiart1ev1aaatCvAUfKttLearuWrP9MDH5MBPbIqV92AaeXatLxBI9gBaebbnrfifHhDYfgasaacH8akY=wiFfYdH8Gipec8Eeeu0xXdbba9frFj0=OqFfea0dXdd9vqai=hGuQ8kuc9pgc9s8qqaq=dirpe0xb9q8qiLsFr0=vr0=vr0dc8meaabaqaciaacaGaaeqabaqabeGadaaakeaat0uy0HwzTfgDPnwy1egaryqtHrhAL1wy0L2yHvdaiqaacqWFce=qaaa@3824@. Finally, one may notice that when *β *→ 0 the exponent of Eq. (17)

−n2〈x2〉y2,
 MathType@MTEF@5@5@+=feaafiart1ev1aaatCvAUfKttLearuWrP9MDH5MBPbIqV92AaeXatLxBI9gBaebbnrfifHhDYfgasaacH8akY=wiFfYdH8Gipec8Eeeu0xXdbba9frFj0=OqFfea0dXdd9vqai=hGuQ8kuc9pgc9s8qqaq=dirpe0xb9q8qiLsFr0=vr0=vr0dc8meaabaqaciaacaGaaeqabaqabeGadaaakeaacqGHsisldaWcaaqaaiabd6gaUbqaaiabikdaYiabgMYiHlabdIha4naaCaaaleqabaGaeGOmaidaaOGaeyOkJepaaiabdMha5naaCaaaleqabaGaeGOmaidaaOGaeiilaWcaaa@39A9@

approaches as expected from central limit theorem.

In the case where the first moment x¯
 MathType@MTEF@5@5@+=feaafiart1ev1aaatCvAUfKttLearuWrP9MDH5MBPbIqV92AaeXatLxBI9gBaebbnrfifHhDYfgasaacH8akY=wiFfYdH8Gipec8Eeeu0xXdbba9frFj0=OqFfea0dXdd9vqai=hGuQ8kuc9pgc9s8qqaq=dirpe0xb9q8qiLsFr0=vr0=vr0dc8meaabaqaciaacaGaaeqabaqabeGadaaakeaacuWG4baEgaqeaaaa@2E3D@ ≡ ∫*xg*(*x*)*dx *≠ 0, one may simply replace *y *by (*y *- x¯
 MathType@MTEF@5@5@+=feaafiart1ev1aaatCvAUfKttLearuWrP9MDH5MBPbIqV92AaeXatLxBI9gBaebbnrfifHhDYfgasaacH8akY=wiFfYdH8Gipec8Eeeu0xXdbba9frFj0=OqFfea0dXdd9vqai=hGuQ8kuc9pgc9s8qqaq=dirpe0xb9q8qiLsFr0=vr0=vr0dc8meaabaqaciaacaGaaeqabaqabeGadaaakeaacuWG4baEgaqeaaaa@2E3D@) in Eq. (17).

## Brief description of implementation

The operation of RAId_DbS consists of three stages. The first step includes centroidizing m/z peaks followed by peak filtering. After this crucial step, RAId_DbS *exhaustively *scores all possible C- and N-tags of four amino acids. This helps RAId_DbS in filtering peptide candidates with NNTC before full scoring. More details for those parts can be found in the appendix. In the third stage, RAId_DbS uses primarily the *b*- and *y*-series peaks for scoring. For each query spectrum, the collection of scores from all candidate peptides constitutes a score histogram, that is then used to determine the constant C
 MathType@MTEF@5@5@+=feaafiart1ev1aaatCvAUfKttLearuWrP9MDH5MBPbIqV92AaeXatLxBI9gBaebbnrfifHhDYfgasaacH8akY=wiFfYdH8Gipec8Eeeu0xXdbba9frFj0=OqFfea0dXdd9vqai=hGuQ8kuc9pgc9s8qqaq=dirpe0xb9q8qiLsFr0=vr0=vr0dc8meaabaqaciaacaGaaeqabaqabeGadaaakeaat0uy0HwzTfgDPnwy1egaryqtHrhAL1wy0L2yHvdaiqaacqWFce=qaaa@3824@ (and other parameters) of theoretical distribution, see Eq. (17). Once all parameters are determined, one then integrates the pdf from infinitely large score back to a finite score S to obtain the spectrum-specific *P*-value for score S. The goodness of the theoretical distribution is then assessed. These information are then used in conjunction with the effective database size to provide the *E*-value.

## Results

**Figure 1:**
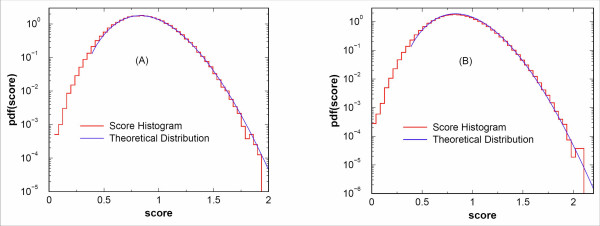
**Comparison of score histogram versus theoretical distribution**. Comparison of score histogram versus theoretical distribution. A randomly picked query spectrum is used to score peptides in NCBI's nr database. For this query spectrum, nine hundred unit intensity peaks were added to the processed spectrum to match S_us_. In panel (A), the red staircase represents the histogram of scores computed using Eq. (1) with *w*_*i *_= 1, while the blue line represents the theoretical distribution predicted from peptides with *n *= 44 theoretical peaks. In panel (B), scores computed using Eq. (1) with *w*_*i*_(*m*_*i*_) = exp(-Δ *m*_*i*_) for peptides with different numbers of theoretical peaks are collected, resulting in the overall score histogram represented by the red staircase. The solid curve plots our fitting of the histogram using Eq. (17) where the fitting variables are *β*, *γ *≡ *n*/(6⟨*x*^2^⟩ *β*^2^) and C
 MathType@MTEF@5@5@+=feaafiart1ev1aaatCvAUfKttLearuWrP9MDH5MBPbIqV92AaeXatLxBI9gBaebbnrfifHhDYfgasaacH8akY=wiFfYdH8Gipec8Eeeu0xXdbba9frFj0=OqFfea0dXdd9vqai=hGuQ8kuc9pgc9s8qqaq=dirpe0xb9q8qiLsFr0=vr0=vr0dc8meaabaqaciaacaGaaeqabaqabeGadaaakeaat0uy0HwzTfgDPnwy1egaryqtHrhAL1wy0L2yHvdaiqaacqWFce=qaaa@3824@.

Eq. (17) is derived for fixed *n *(the number of peaks used to score). Using a random database, if one were to score only peptides with the same number of theoretical peaks, one should be able to obtain the distribution with the overall constant C
 MathType@MTEF@5@5@+=feaafiart1ev1aaatCvAUfKttLearuWrP9MDH5MBPbIqV92AaeXatLxBI9gBaebbnrfifHhDYfgasaacH8akY=wiFfYdH8Gipec8Eeeu0xXdbba9frFj0=OqFfea0dXdd9vqai=hGuQ8kuc9pgc9s8qqaq=dirpe0xb9q8qiLsFr0=vr0=vr0dc8meaabaqaciaacaGaaeqabaqabeGadaaakeaat0uy0HwzTfgDPnwy1egaryqtHrhAL1wy0L2yHvdaiqaacqWFce=qaaa@3824@ as the only fitting parameter. This is tested by using *w*_*i *_= 1 in Eq. (1). In Fig 1(A), we show the score histogram from scoring a query spectrum against peptides within the NCBI's nr database. Only scores from peptides with 44 theoretical peaks are included. Once the score histogram is normalized, we first find S_u_, the highest point of the histogram. The number of unit intensity peaks in the processed/filtered spectrum is then determined by S_u _through S_u _= ⟨ln *I*⟩. All the cumulants are then calculated using the processed/filtered spectrum and the only free parameter left is C
 MathType@MTEF@5@5@+=feaafiart1ev1aaatCvAUfKttLearuWrP9MDH5MBPbIqV92AaeXatLxBI9gBaebbnrfifHhDYfgasaacH8akY=wiFfYdH8Gipec8Eeeu0xXdbba9frFj0=OqFfea0dXdd9vqai=hGuQ8kuc9pgc9s8qqaq=dirpe0xb9q8qiLsFr0=vr0=vr0dc8meaabaqaciaacaGaaeqabaqabeGadaaakeaat0uy0HwzTfgDPnwy1egaryqtHrhAL1wy0L2yHvdaiqaacqWFce=qaaa@3824@. By plotting on a linear-log scale the normalized histogram and the expression in Eq. (17) without including C
 MathType@MTEF@5@5@+=feaafiart1ev1aaatCvAUfKttLearuWrP9MDH5MBPbIqV92AaeXatLxBI9gBaebbnrfifHhDYfgasaacH8akY=wiFfYdH8Gipec8Eeeu0xXdbba9frFj0=OqFfea0dXdd9vqai=hGuQ8kuc9pgc9s8qqaq=dirpe0xb9q8qiLsFr0=vr0=vr0dc8meaabaqaciaacaGaaeqabaqabeGadaaakeaat0uy0HwzTfgDPnwy1egaryqtHrhAL1wy0L2yHvdaiqaacqWFce=qaaa@3824@, one may determine the overall shift log(C
 MathType@MTEF@5@5@+=feaafiart1ev1aaatCvAUfKttLearuWrP9MDH5MBPbIqV92AaeXatLxBI9gBaebbnrfifHhDYfgasaacH8akY=wiFfYdH8Gipec8Eeeu0xXdbba9frFj0=OqFfea0dXdd9vqai=hGuQ8kuc9pgc9s8qqaq=dirpe0xb9q8qiLsFr0=vr0=vr0dc8meaabaqaciaacaGaaeqabaqabeGadaaakeaat0uy0HwzTfgDPnwy1egaryqtHrhAL1wy0L2yHvdaiqaacqWFce=qaaa@3824@) needed through regression. The solid curve in Fig 1(A) is theoretical distribution from Eq. (17) with C
 MathType@MTEF@5@5@+=feaafiart1ev1aaatCvAUfKttLearuWrP9MDH5MBPbIqV92AaeXatLxBI9gBaebbnrfifHhDYfgasaacH8akY=wiFfYdH8Gipec8Eeeu0xXdbba9frFj0=OqFfea0dXdd9vqai=hGuQ8kuc9pgc9s8qqaq=dirpe0xb9q8qiLsFr0=vr0=vr0dc8meaabaqaciaacaGaaeqabaqabeGadaaakeaat0uy0HwzTfgDPnwy1egaryqtHrhAL1wy0L2yHvdaiqaacqWFce=qaaa@3824@ fitted through a least squares procedure.

When scoring peptides against a query spectrum, peptides within the given mass range will not have an identical number of *b *(or *y*) peaks. Separating the candidate peaks into different groups, each with a fixed number of *b *(or *y*) peaks is not practical. Further, we also wish to encourage mass accuracy and thus score candidate peptides using Eq. (1) with weights *w*_*i *_turned on. We still need characterizable statistics even with all of those additional complications. Fortunately, in this case all we need to do is consider *β *and *γ *≡ *n/*(6⟨*x*^2^⟩*β*^2^) as two additional variables to be determined from fitting the score histogram. In Fig. 1(B), using a typical query spectrum, the red staircase is the score histogram with scores from database peptides with allowed molecular weights; each peptide was scored using Eq. (1) with weights *w*_*i*_(*m*_*i*_) = exp(-| Δ *m*_*i*_|). The solid curve is obtained from fitting the histogram with *β*, *γ *and log C
 MathType@MTEF@5@5@+=feaafiart1ev1aaatCvAUfKttLearuWrP9MDH5MBPbIqV92AaeXatLxBI9gBaebbnrfifHhDYfgasaacH8akY=wiFfYdH8Gipec8Eeeu0xXdbba9frFj0=OqFfea0dXdd9vqai=hGuQ8kuc9pgc9s8qqaq=dirpe0xb9q8qiLsFr0=vr0=vr0dc8meaabaqaciaacaGaaeqabaqabeGadaaakeaat0uy0HwzTfgDPnwy1egaryqtHrhAL1wy0L2yHvdaiqaacqWFce=qaaa@3824@ as variables using a least squares procedure. As one may see, the statistics provided using Eq. (17) seems to capture the nature of score distribution reasonably well. The goodness of the fitting to the theoretical distribution may be quantified by a Student's *t*-test. The importance of such a test and its implication will be discussed in detail in the next section.

**Figure 2:**
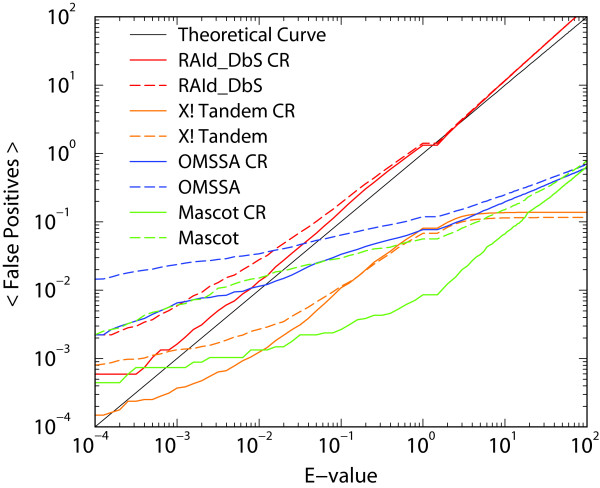
**Average cumulative number of false positives versus *E*-values**. Average cumulative number of false positives versus *E*-values. Theoretically speaking, average number of false positives with *E*-values less than or equal to a cutoff *E*_*c *_should be *E*_*c *_provided that the number of trials is large enough. The accuracy of *E*-values assigned by RAId_DbS is tested along with three other methods, X! Tandem(v1.0), Mascot(v2.1) and OMSSA(v2.0). For X! Tandem, Mascot and OMSSA searches, default parameters of each program are used except the maximum number of miscleavages, which is set to 3 uniformly for this test. The diagonal solid lines in each panel are the theoretical lines. There are two curves associated with each method. The dashed line corresponds to the results using regular nr. The solid line corresponds to the results using nr with cluster removal, which we anticipate to be a better representative of a random database. See text for additional details.

To further test the statistical accuracy of RAId_DbS and a few other search methods reporting *E*-values, we compare the reported *E*-values versus cumulative false positives. The results of the statistical accuracy test are summarized in Fig. 2 and its caption. Two databases are used: the NCBI's nr protein database and nr after cluster removal (CR). CR is done as follows. Each of the eight protein chains is used as a query to search against the NCBI's nr protein database. Proteins hits in nr that align with any of the eight query chains with *E*-values less than 10^-15 ^are removed from the database. This procedure removes 1,848 proteins out of nr which originally contains 1,486,014 proteins.

For a given search method and database, a list of candidate peptides is obtained for every spectrum analyzed. A peptide in the reported list will be classified as a false positive if it is not a subsequence of any of the seven standard proteins used to generate the spectra. For a given *E*-value cutoff, we count cumulatively the total number of false positive peptides assigned with *E*-values less than or equal to that cutoff. Dividing by the total number of spectra, we obtain the average cumulative count of false positives for that *E*-value cutoff. There are in total 6,734 spectra obtained through LTQ/LTQ, LTQ/FT, TOF/TOF, and FT/FT. Therefore, the usable region of this *E*-value accuracy test is limited to *E *≥ 1/6734 ≈ 1.5 × 10^-4^. Fig. 2 shows that RAId_DbS has better statistical accuracy than other methods. In particular, the results for nr after cluster removal seem to reflect well the behavior expected from a random protein database. That is, the resulting curve from RAId_DbS tracks well with the theoretical curve.

Another interesting features of RAId_DbS is that occasionally more than one true positive peptide can be found from the candidate list of a single spectrum without resorting to more elaborate methods such as those of [26]. We first provide an example of this phenomenon and the output format of RAId_DbS. Table 1 displays the output of RAId_DbS using a query spectrum produced by LTQ/LTQ. The output of this spectrum is closely examined because it has multiple low *E*-value peptide hits. Note that the amino acid preceding a peptide's N-terminal is reported along with that peptide. Thus, the first letter in a reported sequence is not to be considered as part of the candidate peptide. The first two peptides reported, therefore, are identical. And the third to fifth peptides reported are also identical if one does not distinguish Leucine from Isoleucine. The significant peptide hits, MYLGYEYVTAIR and LGEYGFQNAILVR, have *E*-values around 4.4 × 10^-5 ^and 1.5 × 10^-4 ^respectively. On the other hand, the third best unique peptide TTLALQFLMEGVR has *E*-value around 1.5, indicating that it is probably a false hit. When the N-terminal of a peptide is actually the N-terminal of a protein, RAId_DbS insert an additional symbol "[" in front of the peptide. An example of such is seen in the last peptide shown in Table 1.

**Table 1:** Example output of RAId_DbS containing multiple significant peptide hits. The contents in the "DEFINITION" and "GI-LIST" columns have been shortened to fit the page. The first two hits correspond to the same peptide MYLGYEYVTAIR, while the third to the fifth hits correspond to the same peptide LGEYGFQNALLVR if we follow the mass spectrometry convention not to distinguish Leucine from Isoleucine. After that, the next peptide has an *E*-value 1.5, indicating a false hit. One thing worth noticing is that there is a clean separation between significant hits and the rest of peptide hits

E-VALUE	PEPTIDE	MASS	DEFINITION	GI-LIST
4.423375e-05	KMYLGYEYVTAIR	1478.720	..|ref|NP 001054.1| transferrin [Homo sapiens]	[4557871,94717618,15021381,31415705,......
4.423375e-05	RMYLGYEYVTAIR	1478.720	..|emb|CAH91543.1| hypothetical protein [Pongo	[55729628]
1.488740e-04	KLGEYGFQNAILVR	1479.780	..|ref|NP 033784.1| albumin 1 [Mus musculus]	[33859506,55391508,191765,19353306, .......
1.488740e-04	KLGEYGFQNALLVR	1479.780	..|emb|CAA59279.1| albumin precursor [Felis	[886485,57977283,633938,30962111, ......
1.488740e-04	KLGEYGFQNALIVR	1479.780	..|gb|AAT98610.1| albumin [Sus scrofa]	[51235682,52353352,15808978,76445989,......
1.526504e+00	KTTLALQFLMEGVR	1478.800	..|ref|YP 466151.1| putative circadian	[86159366]
3.710973e+00	[MFKANMKQLIVR	1478.820	..|dbj|BAD64473.1| cell wall lytic activ	[56909946]
.	.	.	.	.
.	.	.	.	.
.	.	.	.	.

A closer examination shows that both reported significant peptides, MYLGYEYVTAIR and LGEYGFQNAILVR, actually are partial sequences of two of the seven proteins in the mixture. Therefore, it is likely that both peptides are *true positives *co-eluted during the chromatography. On the other hand, if two peptides happen to share a large number of theoretical peaks, then it becomes possible that evidence peaks supporting one peptide will also support the other peptide. In this case, the two peptides may be reported together by accident instead of due to co-elution. To further investigate this possibility, we list in Table 2 the theoretical peaks of both peptides and look for theoretical peaks with similar m/z. It turns out that there is no significant overlap between the *b *∪ *y *peaks from the two peptides. This further supports the possibility that both peptides were co-eluted and good statistical assessment may help us to retain both true positives. Upon analyzing the 6,734 spectra using RAId_DbS, there are 21 spectra each having two true positives with their *E*-values smaller than 10^-2^. There are 93 spectra each having two true positives with their *E*-values smaller than 1.

**Table 2:** Theoretical peaks of two peptides MYLGYEYVTAIR and LGEYGFQNALLVR. Both peptides are found to be significant by RAId_DbS for a given query spectrum and were found to be partial sequences of proteins originally put in for the experiment. The right column lists the *b *∪ *y *peaks of both peptides in ascending m/z order. The two sets of theoretical peaks only have two pairs that are within three daltons of each other. They are (175.12, 175.12) and (1019.45, 1017.58). This negligible overlap between theoretical peaks reinforces the possibility of co-elution of the two peptides during the experiment

Peptide/Mass	*b *∪ *y *peaks (in ascending order)
MYLGYEYVTAIR 1478.72	132.04, 175.12, 288.2, 295.11, 359.24, 408.2, 460.29, 465.22, 559.36, 628.28, 722.42, 757.32, 851.46, 920.39, 1014.53, 1019.45, 1071.55, 1120.5, 1184.63, 1304.62, 1347.69
LGEYGFQNALLVR 1479.79	114.08, 171.11, 175.12, 274.19, 300.16, 387.27, 463.22, 500.36, 520.24, 571.39, 667.31, 685.44, 795.37, 813.38, 90 9.41, 960.56, 980.45, 1017.58, 1093.53, 1180.65, 1206.62, 1305.68, 1309.69, 1366.71

**Figure 3:**
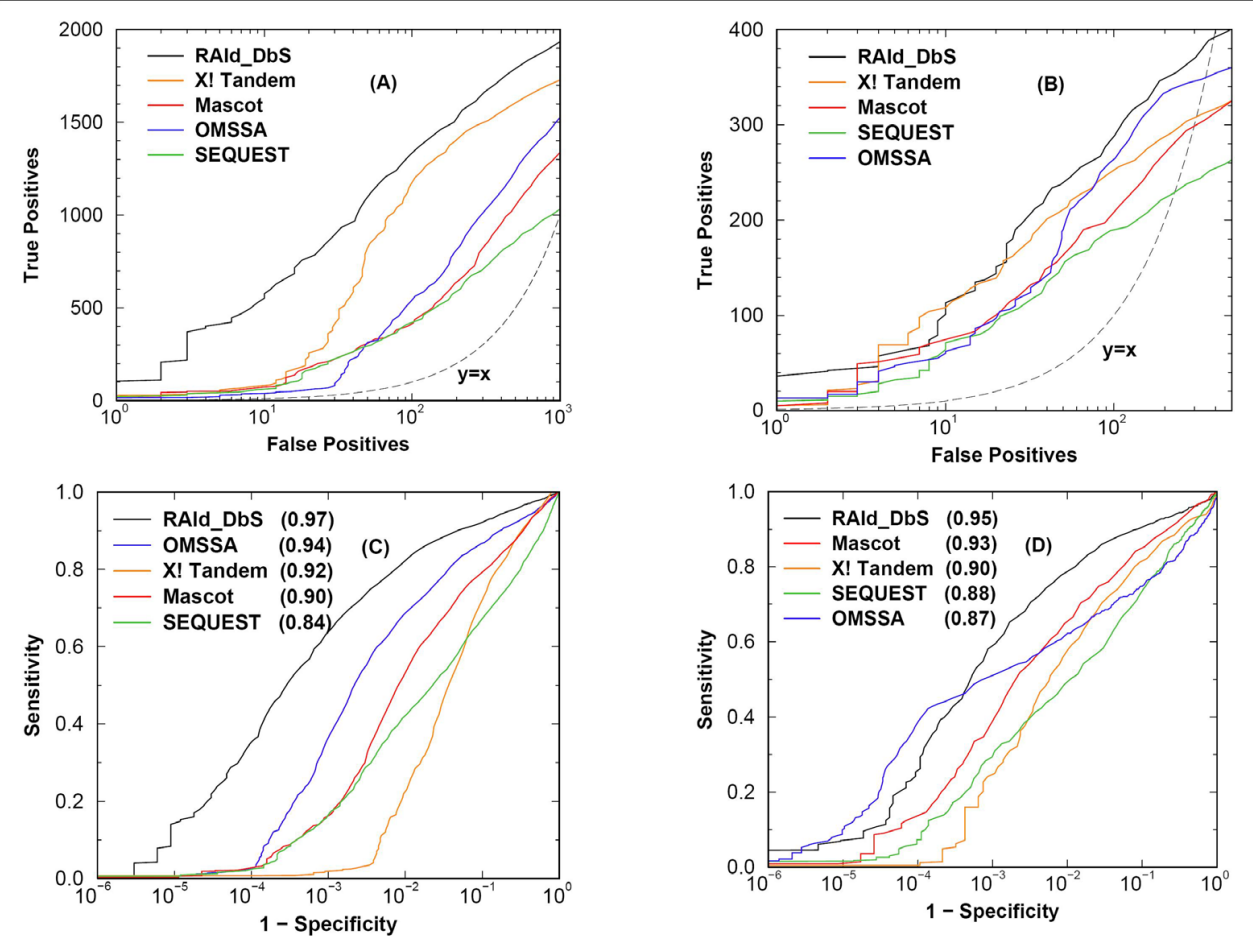
**Performance analysis of methods tested**. Performance analysis of RAId_DbS, X! Tandem(v1.0), Mascot(v2.1), OMSSA(v2.0), and SEQUEST(v3.2). Panels (A) and (C) display the results from 6, 734 spectra in profile format, while panels (B) and (D) display the results from 6,592 centroidized spectra obtained from [19]. In panels (A) and (B), typical ROC curves are shown with the number of false positives (FP) plotted along the abscissa, and the number of true positives (TP) plotted along the ordinate. Thus, a curve that is more to the upper-left corner implies better performance. To unveil the information in the region of small number of false positives, usually the region of most interest, we have plotted the abscissa in log-scale. In panels (C) and (D), a different types of ROC curves are shown. Defining the cumulative number of true negatives by *TN *and the cumulative number of false negative by *FN*, the ROC cuves in panels (C) and (D) plot "1 – specificity" (*FP*/(*FP *+ *TN*)) along the abscissa (also in log-scale), and the sensitivity (*TP*/(*TP *+ *FN*)) along the ordinate. For each method tested, the area under curve (AUC) of this type of ROC curves, when both axes are plotted in linear scale, is also shown inside parentheses in the figure legend. All the AUC have an uncertainty about ± 0.005. Note that ROC curves of this type do not reflect the total number of correct hits and methods that report very few negatives may result in a lower specificity and superficially seems inferior. For example, X! Tandem may be victimized when evaluated using this type of ROC curves. Also note that in panel (D) the trend of AUC for Mascot, X! Tandem, and SEQUEST is consistent with previously reported results [14]. For X! Tandem, Mascot, OMSSA, and SEQUEST, the default parameters for each method were used in every search. However, the maximum number of miscleavages is set to 3 uniformly. It is observed that analysis using profile data giving rise to better ROC curves than those of centoidized data. Although this may be due to the fact that the profile data contain more information, it may also be caused by spectral quality and sample concentration variations.

Finally, we test the effectiveness of RAId_DbS in database retrieval along with several other search methods using Receiver Operating Characteristic (ROC) analysis. The results from spectra with profile (centrodized) format are displayed in panel A (B) of Fig. 3. Although the results in panel (A) seem to suggest that RAId_DbS perform better than X! Tandem and significantly better than other methods, this may be largely due to the fact that RAId_DbS is designed to take the profile data while other methods may not. This is supported by our other assessment using centroidized data published by the Institute for Systems Biology [19]. Data sets A1–A4 of [19] (consisting of 6, 592 spectra) were used for this test. As we may see in panel (B) of Fig. 3, the overall performance gain of RAId_DbS relative to other methods decreases. Nevertheless, this result indicates that by recording the spectrum in profile format, one may have a better chance of uncovering the true peptide(s). Although this may be because the profile data contains more information than centroid data, it may also be caused by spectral quality and sample concentration variations.

## Accuracy of score pdf modeling

To address the accuracy of score pdf modeling, we define two *spectrum-specific *pdfs, *data-derived *pdf (Dpdf) and *model *pdf (Mpdf). For a given query spectrum, the former, Dpdf, is the normalized score histogram including contributions from both the *true positive *peptides and the *false positive *peptides; the latter, Mpdf, represents the pdf of only the *false positives *in the limit of very large number of qualified peptides. For example, we have derived the model Mpdf (eq. (17)) in this paper for the scoring function we used. However, in most cases, the forms of the Mpdf are assumed because analytical results for the Mpdf are difficult to obtain in general.

Ideally, the Mpdf should resemble very much the *re-normalized *score histogram after removing the *true positives*, at least in the region where the fluctuations are negligible compared to the corresponding Mpdf value. At the very high scoring tail, one typically does not have enough data to suppress the fluctuations and there may exist true positives that should not be counted towards Mpdf. Thus, one *cannot *use the tail region of the Dpdf to assign the statistical significance for peptides, an Mpdf extrapolated from high but not very high scoring region is needed for this purpose. This underscores the importance of the accuracy of the Mpdf as it heavily influences the statistical significance assignment. Note that one may wish to have only the large score part modeled faithfully as it is the region of primary interest. However, good agreement between the Dpdf and the Mpdf over a wider range of score does increase the confidence in the validity of the Mpdf. Furthermore, if one were to include a large range of score in Dpdf when fitting to Mpdf, the fluctuations from high scoring tail of Dpdf will not be sufficient to distort the overall Mpdf fit and one may just fit over the entire medium to large score region to obtain the Mpdf.

Because of its importance, for each search engine the accuracy of the Mpdf employed should be reported along with the *E*- or *P*-values for peptide hits when reporting the search results from a query spectrum. For a given query spectrum, if the Mpdf agrees well with the Dpdf, the reported statistics can be taken with confidence. On the other hand, if the agreement between the Mpdf and the Dpdf is poor, one may avoid taking the reported statistics literally. A quantification of fitting quality between the Dpdf and the Mpdf may therefore provide the users with valuable information in data interpretation. In this section, we will attempt to quantify the accuracy of the Mpdf in terms of how well it reflects the Dpdf.

Although there exist standard methods for characterizing the goodness/badness of fitting distribution function, not all of them have similar sensitivity or intuitive appeal. For example, as documented in the literature [27], *χ*^2 ^tests often results in very small goodness numbers even for good models and one often needs to set the rejection threshold very low to avoid rejecting decent models. The Dpdf, derived from the score histogram, is discrete in nature and may be expressed as a list of pairs {S_*i*_, Dpdf(S_*i*_)}_*i *_To emphasize the region of medium score to large score, it is better to work with the log-scale. That is, we will transform the list into {S_*i*_, ln [Dpdf(S_*i*_)]}_*i *_. We introduce a short hand notation here: LDpdf(S) represents ln [Dpdf(S)] and similarly LMpdf(S) represents ln [Mpdf(S)]

The Mpdf, when taking values at {S_*i*_} will also form a list of pairs {S_*i*_, LMpdf(S_*i*_)}_*i*_. If the Mpdf reproduces exactly Dpdf at those points, the pairs Γ ≡ {(LDpdf(S_*i*_), LMpdf(S_*i*_))}_*i *_when plotted on a plane will fall on the straight line *x *= *y *exactly. It is thus natural to ask how well the points in Γ fall on the *x *= *y *line and how strongly are the two sets {LDpdf(S_*i*_)}_*i *_and {LMpdf(S_*i*_)}_*i *_correlated. Fortunately, there exist two Student's *t*-tests that may serve these purposes [28]. We must emphasize that although these two t-tests are useful, there is definitely room for improvement in terms of quantification of the accuracy of Mpdf.

The first t-test concerns how well the data points in Γ fall on the *x *= *y *line. In this case, we have

t1=b−12(N−2)∑i[LDpdf(Si)−LDpdf¯]2∑i[LMpdf(Si)−(a+b LDpdf(Si))]2
 MathType@MTEF@5@5@+=feaafiart1ev1aaatCvAUfKttLearuWrP9MDH5MBPbIqV92AaeXatLxBI9gBaebbnrfifHhDYfgasaacH8akY=wiFfYdH8Gipec8Eeeu0xXdbba9frFj0=OqFfea0dXdd9vqai=hGuQ8kuc9pgc9s8qqaq=dirpe0xb9q8qiLsFr0=vr0=vr0dc8meaabaqaciaacaGaaeqabaqabeGadaaakeaacqWG0baDdaWgaaWcbaGaeGymaedabeaakiabg2da9maalaaabaGaemOyaiMaeyOeI0IaeGymaedabaGaeGOmaidaamaakaaabaWaaSaaaeaacqGGOaakcqWGobGtcqGHsislcqaIYaGmcqGGPaqkdaaeqaqaamaadmaabaGaeeitaWKaeeiraqKaeeiCaaNaeeizaqMaeeOzayMaeiikaGIaee4uam1aaSbaaSqaaiabdMgaPbqabaGccqGGPaqkcqGHsisldaqdaaqaaiabbYeamjabbseaejabbchaWjabbsgaKjabbAgaMbaaaiaawUfacaGLDbaadaahaaWcbeqaaiabikdaYaaaaeaacqWGPbqAaeqaniabggHiLdaakeaadaaeqaqaamaadmaabaGaeeitaWKaeeyta0KaeeiCaaNaeeizaqMaeeOzayMaeiikaGIaee4uam1aaSbaaSqaaiabdMgaPbqabaGccqGGPaqkcqGHsislcqGGOaakcqWGHbqycqGHRaWkcqWGIbGycqqGGaaicqqGmbatcqqGebarcqqGWbaCcqqGKbazcqqGMbGzcqGGOaakcqqGtbWudaWgaaWcbaGaemyAaKgabeaakiabcMcaPiabcMcaPaGaay5waiaaw2faamaaCaaaleqabaGaeGOmaidaaaqaaiabdMgaPbqab0GaeyyeIuoaaaaaleqaaaaa@7404@

with LDpdf¯
 MathType@MTEF@5@5@+=feaafiart1ev1aaatCvAUfKttLearuWrP9MDH5MBPbIqV92AaeXatLxBI9gBaebbnrfifHhDYfgasaacH8akY=wiFfYdH8Gipec8Eeeu0xXdbba9frFj0=OqFfea0dXdd9vqai=hGuQ8kuc9pgc9s8qqaq=dirpe0xb9q8qiLsFr0=vr0=vr0dc8meaabaqaciaacaGaaeqabaqabeGadaaakeaadaqdaaqaaiabbYeamjabbseaejabbchaWjabbsgaKjabbAgaMbaaaaa@32F4@ representing the average of the set {LDpdf(S_*i*_)}_*i*_, *a *and *b *being respectively the intercept and the slope obtained from least square linear regression of Γ, *N *being the number paired points included in Γ. The goodness of the assumption -points fall on *x *= *y *line- may be expressed as 1 *- A*(*t*_1_|*N *- 2) with

A(t|ν)≡1ν1/2B(12,ν2)∫−|t||t|(1+x2ν)−ν+12dx
 MathType@MTEF@5@5@+=feaafiart1ev1aaatCvAUfKttLearuWrP9MDH5MBPbIqV92AaeXatLxBI9gBaebbnrfifHhDYfgasaacH8akY=wiFfYdH8Gipec8Eeeu0xXdbba9frFj0=OqFfea0dXdd9vqai=hGuQ8kuc9pgc9s8qqaq=dirpe0xb9q8qiLsFr0=vr0=vr0dc8meaabaqaciaacaGaaeqabaqabeGadaaakeaacqWGbbqqcqGGOaakcqWG0baDcqGG8baFiiGacqWF9oGBcqGGPaqkcqGHHjIUdaWcaaqaaiabigdaXaqaaiab=17aUnaaCaaaleqabaGaeGymaeJaei4la8IaeGOmaidaaOGaemOqai0aaeWaaeaadaWcaaqaaiabigdaXaqaaiabikdaYaaacqGGSaaldaWcaaqaaiab=17aUbqaaiabikdaYaaaaiaawIcacaGLPaaaaaWaa8qmaeaadaqadaqaaiabigdaXiabgUcaRmaalaaabaGaemiEaG3aaWbaaSqabeaacqaIYaGmaaaakeaacqWF9oGBaaaacaGLOaGaayzkaaaaleaacqGHsisldaabdaqaaiabdsha0bGaay5bSlaawIa7aaqaamaaemaabaGaemiDaqhacaGLhWUaayjcSdaaniabgUIiYdGcdaahaaWcbeqaaiabgkHiTmaalaaabaGae8xVd4Maey4kaSIaeGymaedabaGaeGOmaidaaaaakiabdsgaKjabdIha4baa@602C@

where *B*(*α*, *ν*) is the Beta function. This measure of goodness is intuitive and will allow the user to set a cutoff to prevent from using corrupted fitting results. We suggest to accept the Mpdf only if the goodness number is larger than 0.1. This should be contrasted with popular *χ*^2 ^test where setting a goodness threshold at 10^-3 ^or smaller is common [27].

Once we accept the Mpdf, we also need to know to what degree does our fitted Mpdf represent the *true *pdf comprised of a large number of false peptides. To quantify the accuracy of the Mpdf, we first calculate the correlation strength between {LDpdf(S_*i*_)}_*i *_and {LMpdf(S_*i*_)}_*i*_. In general, the correlation *r *between those two sets may be written as

r=∑i[LDpdf(Si)−LDpdf¯][LMpdf(Si)−LMpdf¯][∑i(LDpdf(Si)−LDpdf¯)2]1/2[∑i(LMpdf(Si)−LMpdf¯)2]1/2,
 MathType@MTEF@5@5@+=feaafiart1ev1aaatCvAUfKttLearuWrP9MDH5MBPbIqV92AaeXatLxBI9gBaebbnrfifHhDYfgasaacH8akY=wiFfYdH8Gipec8Eeeu0xXdbba9frFj0=OqFfea0dXdd9vqai=hGuQ8kuc9pgc9s8qqaq=dirpe0xb9q8qiLsFr0=vr0=vr0dc8meaabaqaciaacaGaaeqabaqabeGadaaakeaacqWGYbGCcqGH9aqpdaWcaaqaamaaqababaWaamWaaeaacqqGmbatcqqGebarcqqGWbaCcqqGKbazcqqGMbGzcqGGOaakcqqGtbWudaWgaaWcbaGaemyAaKgabeaakiabcMcaPiabgkHiTmaanaaabaGaeeitaWKaeeiraqKaeeiCaaNaeeizaqMaeeOzaygaaaGaay5waiaaw2faamaadmaabaGaeeitaWKaeeyta0KaeeiCaaNaeeizaqMaeeOzayMaeiikaGIaee4uam1aaSbaaSqaaiabdMgaPbqabaGccqGGPaqkcqGHsisldaqdaaqaaiabbYeamjabb2eanjabbchaWjabbsgaKjabbAgaMbaaaiaawUfacaGLDbaaaSqaaiabdMgaPbqab0GaeyyeIuoaaOqaamaadmaabaWaaabeaeaacqGGOaakcqqGmbatcqqGebarcqqGWbaCcqqGKbazcqqGMbGzcqGGOaakcqqGtbWudaWgaaWcbaGaemyAaKgabeaakiabcMcaPiabgkHiTmaanaaabaGaeeitaWKaeeiraqKaeeiCaaNaeeizaqMaeeOzaygaaiabcMcaPmaaCaaaleqabaGaeGOmaidaaaqaaiabdMgaPbqab0GaeyyeIuoaaOGaay5waiaaw2faamaaCaaaleqabaGaeGymaeJaei4la8IaeGOmaidaaOWaamWaaeaadaaeqaqaaiabcIcaOiabbYeamjabb2eanjabbchaWjabbsgaKjabbAgaMjabcIcaOiabbofatnaaBaaaleaacqWGPbqAaeqaaOGaeiykaKIaeyOeI0Yaa0aaaeaacqqGmbatcqqGnbqtcqqGWbaCcqqGKbazcqqGMbGzaaGaeiykaKYaaWbaaSqabeaacqaIYaGmaaaabaGaemyAaKgabeqdcqGHris5aaGccaGLBbGaayzxaaWaaWbaaSqabeaacqaIXaqmcqGGVaWlcqaIYaGmaaaaaOGaeiilaWcaaa@94F1@

and the corresponding *t *variable may be expressed as

t2=rν1−r2
 MathType@MTEF@5@5@+=feaafiart1ev1aaatCvAUfKttLearuWrP9MDH5MBPbIqV92AaeXatLxBI9gBaebbnrfifHhDYfgasaacH8akY=wiFfYdH8Gipec8Eeeu0xXdbba9frFj0=OqFfea0dXdd9vqai=hGuQ8kuc9pgc9s8qqaq=dirpe0xb9q8qiLsFr0=vr0=vr0dc8meaabaqaciaacaGaaeqabaqabeGadaaakeaacqWG0baDdaWgaaWcbaGaeGOmaidabeaakiabg2da9iabdkhaYnaakaaabaWaaSaaaeaaiiGacqWF9oGBaeaacqaIXaqmcqGHsislcqWGYbGCdaahaaWcbeqaaiabikdaYaaaaaaabeaaaaa@3800@

with *ν *being the number of points in Γ less the number of fitting parameters of the Mpdf. The probability to arrive at correlation *r*, assuming that {Dpdf(S_*i*_)}_*i *_and {Mpdf(S_*i*_)}_*i *_are drawn from random, is given by

*P*_*M *_= 1 - *A*(*t*_2_|*ν*).

In a way, *P*_*M *_may also be viewed as the probability that the Mpdf to be wrong. This observation has a nontrivial consequence in assigning statistical significance to peptide hits. It sets a limit on the lowest *P*-value one can get for a peptide hit, which we elaborate below.

If we have full confidence in a Mpdf, for a given peptide with score S, one may infer from the Mpdf a *P*-value (and consequently an *E*-value) for this hit. However, if our confidence in the Mpdf is not 100 percent, the statistics reported by the Mpdf may need adjustment. We propose below a simple way to do so. Let the *P*-value reported by the Mpdf for a peptide hit be *P*_*h*_, one may then view 1 - *P*_*h *_as the probability of correct identification. We may also view 1 - *P*_*M *_as the probability for the Mpdf to be correct. Thus, the probability of correct identification confidently supported by the Mpdf becomes (1 - P_*h*_)(1 - *P*_*M*_). And the final *P *-value becomes

*P*_*h*|*M *_= 1 - (1 - *P*_*h*_)(1 - *P*_*M*_) = *P*_*h *_+ *P*_*M *_- *P*_*h*_*P*_*M*_.

Apparently, when *P*_*M *_approaches zero, that is, we have full confidence in the Mpdf, the final *P*-value reduces to *P*_*h*_. As an example of how this formulation may prevent exaggerated statistics, let us consider the case where *P*_*h *_= 10^-50 ^and *P*_*M *_= 10^-8^. Without eq. (23), one will infer a hit of very small *P*-value (10^-50^). With eq. (23), we find that the final *P*-value, 10^-50 ^+ 10^-8 ^- 10^-58 ^= 10^-8 ^+ 10^-50^(1 - 10^-8^), to be greater than 10^-8^. That is, one will not get a smaller *P*-value than *P*_*M*_.

However, one has to pay attention to that 1 - *P*_*h*|*M *_represents the probability of *correct identification supported by confident *Mpdf. It is definitely possible that a method may identify the true peptide as the top hit but the Mpdf used may be very off. However, if this happens frequently for a given search engine, then it becomes hard to pool its search results due to the lack of a common statistical standard. That is, one can't set *a priori *an *E*-value cutoff that should represent the expected number of false positives found per spectrum. If one were to take just the top hit from each spectrum, depending on the spectral quality, one may ended up having many more true/false positives in one experiment than the others.

To provide an example of computing the goodness number for Mpdf and *P*_*M*_, we randomly pick a spectrum with the corresponding data given in Table 3. In each of the *N *= 28 numerical rows of Table 3, the first entry is the score, the second entry records the LDpdf and the third entry corresponds to the LMpdf. Using the LDpdf as the *x*-coordinate and the Mpdf as the *y*-coordinate, we plot the LDpdf versus the LMpdf on the *x*-*y *plane. A least square linear regression give rise to an intercept value *a *= -0.00421 and a slope *b *= 0.9992. With the constants *a *and *b *identified, one may then use (18) to compute *t*_1 _and find the goodness number, 1 - *A*(*t*_1_|*N *- 2), through (19). We find that *t*_1 _= 0.0421 and the goodness number is 0.96674. To test the strength of correlation between the second column and the third column of Table 3, we use (20) to compute the *r *value and through (21) we find the *t*_2 _value to be 0.99567. Given *r *= 0.99567 and *ν *= 25, through (22) we find the *P*_*M *_value to be 2.58 × 10^-27^.

**Table 3:** An example for computing fitting confidence. A randomly chosen spectrum is used to demonstrate the computation of the fitting confidence in detail. In each of the *N *= 28 numerical rows, the first entry is the score, the second entry records the LDpdf and the third entry corresponds to the LMpdf. Using the LDpdf as the *x*-coordinate and the Mpdf as the *y*-coordinate, we perform least square linear regression and find: an intercept value *a *= -0.00421 and a slope *b *= 0.9992. Eq. (18) is then used to compute *t*_1 _(*t*_1 _= 0.0421) and the goodness number, 1 - *A*(*t*_1_|*N *-2), is found to be 0.96674 through (19). To test the strength of correlation between the second column and the third column, we use (20) to compute *r *and through (21) we find the *t*_2 _value to be 0.99567. Given *r *= 0.99567 and = 25, through (22) we find the *P*_*M *_value to be 2.58 × 10^-27.^

S	ln [Dpdf(S)]	ln [Mpdf(S)]
0.0284661	0.479518	0.438266
0.0691319	0.431753	0.407608
0.109798	0.369235	0.351511
0.150463	0.2708	0.270076
0.191129	0.163419	0.163403
0.231795	0.014358	0.031592
0.272461	-0.156812	-0.125259
0.313127	-0.340242	-0.307054
0.353792	-0.551264	-0.513698
0.394458	-0.79275	-0.745095
0.435124	-1.04746	-1.00115
0.47579	-1.34063	-1.28178
0.516456	-1.63587	-1.58688
0.557121	-1.96251	-1.91636
0.597787	-2.2322	-2.27015
0.638453	-2.72001	-2.64814
0.679119	-3.00809	-3.05025
0.719785	-3.52319	-3.4764
0.76045	-3.94211	-3.92649
0.801116	-4.31754	-4.40045
0.841782	-4.72005	-4.89819
0.882448	-5.27305	-5.41962
0.923114	-5.73387	-5.96467
0.963779	-7.04955	-6.53326
1.00445	-6.55707	-7.1253
1.04511	-7.368	-7.74071
1.08578	-9.44744	-8.37942
1.12644	-8.75429	-9.04134

**Figure 4:**
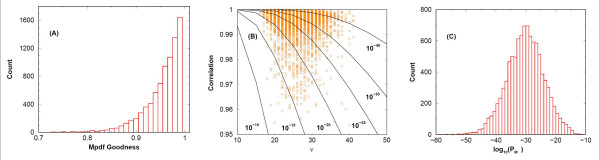
**Quantification of goodness of score model used for statistical significance assignment**. A global study of the Mpdf accuracy using 10,000 spectra (profile mode). Panel (A) shows the histogram of the goodness number. Panel (B) shows a scattered plot of *ν *versus *r *obtained from our spectra as well as a number of curves each corresponds to a fixed *P*_*M *_value. Panel (C) displays the histogram of log_10_(*P*_*M*_).

A global study of the Mpdf accuracy using 10, 000 spectra (profile mode) is summarized in Fig. 4. Panel (A) shows the histogram of the goodness number, panel (B) shows a scattered plot of *ν *versus *r *obtained from our spectra, and panel (C) displays the histogram of log_10_(*P*_*M*_). Also displayed in panel (B) are curves with fixed *P*_*M *_values. As we may see from these plots, the fitting quality of the LDpdf to our theoretical distribution is generally very good. The important message, however, is that each search method should provide the goodness of fitting so that the users can be informed and can decide whether to take the reported statistics seriously or not. We have suggested a goodness number cutoff 0.1 for accepting an Mpdf. The user, however, may choose a slightly larger number as the cutoff to reject Mpdfs that (s)he has less confidence in. As for *P*_*M*_, it is *not necessary *to employ a cutoff there. This is because a poor(large) *P*_*M *_will automatically make any hits found insignificant through eq. (23).

## Concluding summary and outlook

We have designed a peptide identification method (RAId_DbS) using database searches. By taking into account the skewness in the peak intensity distribution of processed data, we have provided a theoretical derivation for the tail of the score distribution in the context of RAId_DbS's scoring scheme. The theoretical distribution agrees well with score statistics collected from each experimental spectrum. The *E*-value test performed indicates that RAId_DbS indeed provides realistic statistics. Quantitative tests on the agreement of our theoretical distribution and data-derived histogram have shown that RAId_DbS assigns accurate *spectrum-specific *statistical significance to peptide hits. The *P*-value obtained through (23) prevents exaggerated statistics in peptide identification, and thus may reduce protein misidentification for identification methods founded on peptide identification.

It seems that using RAId_DbS allows for theoretically characterized peptide score statistics without losing sensitivity, see Fig. 3. In addition, the use of profile mode in data acquisition seems to be valuable because of the higher probability of correct peptide identification. We have also found evidence that during an experiment it is possible for two charged peptides to be co-eluted and fragmented together, and their m/z peaks are logged in a *mixed *spectrum. In this context, RAId_DbS seems to be able to identify both peptides. This phenomenon actually discourages the use of heuristics that boost the separation between the best and the second best candidate peptides. This is because any method attempting such heuristics may be deprived of the possibility to capture two true peptides in a single spectrum.

Finally, we would like to say that there is room for improvement in RAId_DbS. For example, in the future, we would like to improve on the scoring scheme to enhance the sensitivity of RAId_DbS while keeping the characterizable statistics. In addition to improving the detecting power of RAId_DbS, we will also look at the possibility of combining RAId_DbS with other search methods. However, to be able to appropriately combine results from different methods, it is essential to build a common ground for score statistics. This important task will be performed and will be described in a separate publication.

## Appendix – RAId_DbS implementation detail

The operation of RAId_DbS consists of three stages. The first step includes centroidizing m/z peaks followed by peak filtering. After this crucial step, RAId_DbS *exhaustively *scores all possible C- and N-tags of four amino acids. This helps RAId_DbS in filtering peptide candidates with NNTC before full scoring. In the third stage, as in many other MS^2 ^analysis methods (be they the *de novo *type or database search type), RAId_DbS uses primarily the *b*- and *y*-series peaks for scoring. For each query spectrum, the collection of scores from all candidate peptides constitutes a score histogram, that is then used to determine the constant C
 MathType@MTEF@5@5@+=feaafiart1ev1aaatCvAUfKttLearuWrP9MDH5MBPbIqV92AaeXatLxBI9gBaebbnrfifHhDYfgasaacH8akY=wiFfYdH8Gipec8Eeeu0xXdbba9frFj0=OqFfea0dXdd9vqai=hGuQ8kuc9pgc9s8qqaq=dirpe0xb9q8qiLsFr0=vr0=vr0dc8meaabaqaciaacaGaaeqabaqabeGadaaakeaat0uy0HwzTfgDPnwy1egaryqtHrhAL1wy0L2yHvdaiqaacqWFce=qaaa@3824@ of theoretical distribution, see Eq. (17). Once C
 MathType@MTEF@5@5@+=feaafiart1ev1aaatCvAUfKttLearuWrP9MDH5MBPbIqV92AaeXatLxBI9gBaebbnrfifHhDYfgasaacH8akY=wiFfYdH8Gipec8Eeeu0xXdbba9frFj0=OqFfea0dXdd9vqai=hGuQ8kuc9pgc9s8qqaq=dirpe0xb9q8qiLsFr0=vr0=vr0dc8meaabaqaciaacaGaaeqabaqabeGadaaakeaat0uy0HwzTfgDPnwy1egaryqtHrhAL1wy0L2yHvdaiqaacqWFce=qaaa@3824@ is determined, one then integrates the pdf from infinitely large score back to a finite score S to obtain the spectrum-specific *P*-value for score S. This information is then used in conjunction with the effective database size to provide the *E*-value. In the following subsections, we describe each individual component, the sum of which constitutes RAId_DbS, followed by some details of implementation.

### Peak processing

Peak processing can be roughly divided into three steps. In first step, precursor ion peaks and their associated *one-dalton-cluster *ions are removed from spectrum data. One-dalton-cluster ions associated with a peptide fragment of mass *m' *are members of a list of ions having masses given by {*m' *+ *Hyd, m' *+ 2*Hyd*, ...}, with *Hyd *being the mass of hydrogen (1.007825035 Da). For a parent peptide with mass *m *and charge *q*, the precursor and cluster ion peaks to be removed from the spectrum are those having their mass/charge peaks within 0.05 Da of [*m *+ (*q*_*i *_- 1 + *k*) × *Hyd*]/*q*_*i *_for every *q*_*i *_= 1, ..., *q*, and *k *= 0, 1, ..., *q*_*i *_- 1.

Peak centroidizing is the second step of RAId_DbS's peak processing. In the centroidizing procedure, RAId_DbS first identifies what we term *ε*-clusters, then distills from each cluster either a single or multiple representative peaks depending on the noise level that we shall define shortly. An *ε*-cluster consists of a list of peaks, ordered according to their m/z values, for which any two neighboring elements have m/z difference no more than *ε *Da. The *ε *value usually depends on the instrument type used. The current default for *ε *value is 0.2 Da for low resolution spectra such as those produced from Linear Quadrupole Ion Trap (LTQ)/LQT experiment and is 0.05 Da for high resolution spectra such as those produced from Time of Flight (TOF)/TOF or Fourier Transform (FT)/FT. The noise level is currently defined heuristically. For each *ε*-cluster of p_*ε *_peaks, RAId_DbS uses the least intense 2p_*ε*_/3 peaks to compute the average intensity as well as the standard deviations. The noise level is then defined as the average intensity plus three standard deviations. A separate subcluster (a *hill*) is a subsequence of peaks whose intensities are greater than the noise level. Each subcluster is transformed to a separate peak: with m/z at the center of mass of the subcluster, and with intensity being the intensity of the strongest peak in the subcluster. The m/z peaks inside an *ε*-cluster with intensities less than the noise level are disregarded. When there are no hills present in an *ε*-cluster, one treat that *ε*-cluster as a single hill. This step is rather heuristic: we are still investigating possible avenues to improve this.

The third step is peak filtering. The idea is to keep only a finite number of informative peaks within a specified mass range, say ± *x *Da, regardless of where the center is. To be specific, RAId_DbS orders all the peaks produced from the centroidizing steps in two ways: in descending order of intensities and in ascending order of m/z. Going first to the strongest peak, RAId_DbS first makes sure that within 2*ε *Da, only one peak is retained. After that, depending on the charge state of the parent ion, RAId_DbS uses either *x *= 27 for single and doubly charged precursor ions or *x *= 27/(*q *- 1) for precursor ion with charge state *q *≥ 3. RAId_DbS further normalize the peak intensity by a user-selected cutoff *I*_*c*_. Each peak intensity will then be multiplied by 1/*I*_*c *_and m/z peaks with normalized intensities less than one are removed. The current default is *I*_*c *_= 1. That is, no rescaling of the peak intensities.

### *De novo *tag scoring

Besides allowing for any number of miscleavages, we also designed RAId_DbS to accommodate NNTC [17]. Allowing NNTC, however, introduces a huge excess number of peptides to be scored when searching in a database. In order to filter out peptides with higher chance to be the correct peptide, we implement a full *de novo *tag scoring to rank all possible *de novo *tags and only allow peptides with a high-scoring tag to enter scoring routine provided that the peptide considered has NNTC.

Using sequence tags to aid peptide identification is not a new concept. There exist, for example, several known methods [10,29] that use sequence tags to mine candidate peptides in a database. Our use of sequence tags is distinguished from other methods by the following points. First, our sequence tag is used for the purpose of filtering out potential peptide candidates with NNTC [17], not used as a criterion for pooling candidate peptides. Second, for each spectrum we score *all *possible four amino acid tags (20^4 ^for each terminal) and we keep many more tags, of order several thousands for each terminal, when compared with other tag-based method. Another reason for us to score tags is to provide a different foundation for *de novo *peptide sequencing using low resolution data. This direction, however, will be addressed in a separate publication.

All possible four amino acid tags are generated on the fly and scored (see scoring section of the paper for details) using m/z peaks after peak processing. RAId_DbS then ranks all the tags according to their score. However, it should be noted that in some low resolution experiments, the parent ion mass of a peptide reported by a mass analyzer can be as far off as two Da. To tolerate such a mass uncertainty, RAId_DbS actually scores each tag seven times, assuming the parent ion mass to be respectively -*m*_*E *_- 3, *m*_*E *_- 2, *m*_*E *_- 1, *m*_*E*_, *m*_*E*_, + 1, *m*_*E *_+ 2, *m*_*E *_+ 3- with *m*_*E *_being the parent ion mass provided by the mass analyzer from experiment. High-scoring tags, from each of the seven parent ion mass used, are pooled together to form two separate tag lists: one for each terminal. Note that it is possible that the highest scoring N-terminal tag is obtained by assuming parent ion mass to be *m*_*E *_+ 2 while the second best N-terminal tag is obtained by assuming parent ion mass to be *m*_*E *_-3, etc. With care, RAId_DbS can achieve this task in a few seconds.

### Statistical assessment and implementation

For a given MS^2 ^spectrum, RAId_DbS first scores all the possible *de novo *tags as described earlier. This step provides two high-scoring tag lists, one for C-terminal and one for N-terminal. After the tag scoring is done, RAId_DbS scans either a user-chosen or the default protein database for peptides with correct C-terminal cleavage and with matching molecular weights within 3 Da. When a qualified peptide appears multiple times while scanning through the database, RAId_DbS will combine them and only score the peptide once. A peptide with correct N-terminal cleavage will be automatically scored regardless of how many miscleavages are present. On the other hand, peptides with NNTC will be scored only if they contain a high-scoring tag, either from C-terminal or from N-terminal.

The statistics of the peptide scores are collected while scoring each peptide. Ideally, one would like to construct a score histogram for all unique database peptides whose molecular weights fall in the correct mass range, determined by the experimental value and user-defined mass error tolerance. In reality, it could be too time-consuming if we were to do this for all peptides including those with NNTC. Consequently, for peptides with NNTC we only include their scores in the histogram if they have at least one good tag score. While scoring candidate peptides for a query spectrum, RAId_DbS advances counters *U*_*c*_(*k*) and *U*_*n*_(*k*) in the fashion that will be explained below. When a unique peptide with correct N-terminal cleavage and with *k *miscleavages is scored, we advance the counter *U*_*c*_(*k*) by one. Similarly, we advance the counter *U*_*n*_(*k*) by one when a unique peptide with *k *miscleavages and with NNTC is scored. The counter *U*_*n*_(*k*), however, does not include those with poor tag scores. Since to compute the number of miscleavages for all peptides with NNTC would be too time consuming, we keep an additional global counter *G*_*n *_for the total number of database peptides (with either good or bad tag scores) with NNTC and whose molecular weights fall within the right range. To better estimate the total number of unique peptides with *k *miscleavages and with NNTC, we also introduce temporary counter *L*_*n*_(*k*). Basically, every unique peptide contribute one count to *U*_*n*_(*k*) will contribute to *L*_*n*_(*k*) the number of occurrence of that peptide in the database. That is, *L*_*n*_(*k*) contains all the redundancy of *U*_*n*_(*k*). Given a molecular weight range, the total number of peptides with NNTC and with *k *miscleavages is then estimated by

U˜n(k)≡Un(k)Ln(k)[Ln(k)∑k′Ln(k′)]×Gn.
 MathType@MTEF@5@5@+=feaafiart1ev1aaatCvAUfKttLearuWrP9MDH5MBPbIqV92AaeXatLxBI9gBaebbnrfifHhDYfgasaacH8akY=wiFfYdH8Gipec8Eeeu0xXdbba9frFj0=OqFfea0dXdd9vqai=hGuQ8kuc9pgc9s8qqaq=dirpe0xb9q8qiLsFr0=vr0=vr0dc8meaabaqaciaacaGaaeqabaqabeGadaaakeaacuWGvbqvgaacamaaBaaaleaacqWGUbGBaeqaaOGaeiikaGIaem4AaSMaeiykaKIaeyyyIO7aaSaaaeaacqWGvbqvdaWgaaWcbaGaemOBa4gabeaakiabcIcaOiabdUgaRjabcMcaPaqaaiabdYeamnaaBaaaleaacqWGUbGBaeqaaOGaeiikaGIaem4AaSMaeiykaKcaamaadmaabaWaaSaaaeaacqWGmbatdaWgaaWcbaGaemOBa4gabeaakiabcIcaOiabdUgaRjabcMcaPaqaamaaqababaGaemitaW0aaSbaaSqaaiabd6gaUbqabaaabaGafm4AaSMbauaaaeqaniabggHiLdGccqGGOaakcuWGRbWAgaqbaiabcMcaPaaaaiaawUfacaGLDbaacqGHxdaTcqWGhbWrdaWgaaWcbaGaemOBa4gabeaakiabc6caUaaa@56B7@

However, including only peptides with NNTC and good tag score tends to induce more occurrences of high-scoring hits with NNTC than would normally have occurred if one were to score all the peptides with NNTC. This may assign high-scoring peptides with NNTC *P*-values that are too small. Consequently, it is possible that peptides with NNTC may be assigned *E*-values that are too small. Using

U˜n(k)≡[Un(k)∑k′Un(k′)]×Gn
 MathType@MTEF@5@5@+=feaafiart1ev1aaatCvAUfKttLearuWrP9MDH5MBPbIqV92AaeXatLxBI9gBaebbnrfifHhDYfgasaacH8akY=wiFfYdH8Gipec8Eeeu0xXdbba9frFj0=OqFfea0dXdd9vqai=hGuQ8kuc9pgc9s8qqaq=dirpe0xb9q8qiLsFr0=vr0=vr0dc8meaabaqaciaacaGaaeqabaqabeGadaaakeaacuWGvbqvgaacamaaBaaaleaacqWGUbGBaeqaaOGaeiikaGIaem4AaSMaeiykaKIaeyyyIO7aamWaaeaadaWcaaqaaiabdwfavnaaBaaaleaacqWGUbGBaeqaaOGaeiikaGIaem4AaSMaeiykaKcabaWaaabeaeaacqWGvbqvdaWgaaWcbaGaemOBa4gabeaakiabcIcaOiqbdUgaRzaafaGaeiykaKcaleaacuWGRbWAgaqbaaqab0GaeyyeIuoaaaaakiaawUfacaGLDbaacqGHxdaTcqWGhbWrdaWgaaWcbaGaemOBa4gabeaaaaa@4A46@

has the advantage of over estimating the effective database size for peptides with NNTC to compensate for the excessively small *P*-values. This may provide more accurate *E*-values for peptides with NNTC and good tag score. We leave the use of Eq. (25) as an option while keeping Eq. (24) as the default of RAId_DbS.

When fitting the score histogram by Eq. (17), one needs to replace the variable *y *by [S - ⟨lnℐ
 MathType@MTEF@5@5@+=feaafiart1ev1aaatCvAUfKttLearuWrP9MDH5MBPbIqV92AaeXatLxBI9gBaebbnrfifHhDYfgasaacH8akY=wiFfYdH8Gipec8Eeeu0xXdbba9frFj0=OqFfea0dXdd9vqai=hGuQ8kuc9pgc9s8qqaq=dirpe0xb9q8qiLsFr0=vr0=vr0dc8meaabaqaciaacaGaaeqabaqabeGadaaakeaat0uy0HwzTfgDPnwy1egaryqtHrhAL1wy0L2yHvdaiqaacqWFqessaaa@3768@⟩]. However, the quantity ⟨lnℐ
 MathType@MTEF@5@5@+=feaafiart1ev1aaatCvAUfKttLearuWrP9MDH5MBPbIqV92AaeXatLxBI9gBaebbnrfifHhDYfgasaacH8akY=wiFfYdH8Gipec8Eeeu0xXdbba9frFj0=OqFfea0dXdd9vqai=hGuQ8kuc9pgc9s8qqaq=dirpe0xb9q8qiLsFr0=vr0=vr0dc8meaabaqaciaacaGaaeqabaqabeGadaaakeaat0uy0HwzTfgDPnwy1egaryqtHrhAL1wy0L2yHvdaiqaacqWFqessaaa@3768@⟩ may not match ⟨ln *I*⟩ in our processed data. Nevertheless, the exponent in Eq. (17) is a decreasing function for *y *≥ 0 as is evident from

∂∂y{[1−1+2βy][1+4βy−1+2βy]}=6β[1−1+2βy]<0
 MathType@MTEF@5@5@+=feaafiart1ev1aaatCvAUfKttLearuWrP9MDH5MBPbIqV92AaeXatLxBI9gBaebbnrfifHhDYfgasaacH8akY=wiFfYdH8Gipec8Eeeu0xXdbba9frFj0=OqFfea0dXdd9vqai=hGuQ8kuc9pgc9s8qqaq=dirpe0xb9q8qiLsFr0=vr0=vr0dc8meaabaqaciaacaGaaeqabaqabeGadaaakeGacaaU=daxbuaabaqaceaaaeaadaWcaaqaaiabgkGi2cqaaiabgkGi2kabdMha5baadaGadaqaamaadmaabaGaeGymaeJaeyOeI0YaaOaaaeaacqaIXaqmcqGHRaWkcqaIYaGmiiGacqWFYoGycqWG5bqEaSqabaaakiaawUfacaGLDbaadaWadaqaaiabigdaXiabgUcaRiabisda0iab=j7aIjabdMha5jabgkHiTmaakaaabaGaeGymaeJaey4kaSIaeGOmaiJae8NSdiMaemyEaKhaleqaaaGccaGLBbGaayzxaaaacaGL7bGaayzFaaaabiqacGOdcaWLjaGaeyypa0JaeGOnayJae8NSdi2aamWaaeaacqaIXaqmcqGHsisldaGcaaqaaiabigdaXiabgUcaRiabikdaYiab=j7aIjabdMha5bWcbeaaaOGaay5waiaaw2faaiabgYda8iabicdaWaaaaaa@5E8B@

provided that *β *> 0 and *y *≥ 0, the situation we encounter here. Consequently, Eq. (17) dictates that the maximum of the histogram occurs at *y *= 0, corresponding to S_*u *_= ⟨ln ℐ
 MathType@MTEF@5@5@+=feaafiart1ev1aaatCvAUfKttLearuWrP9MDH5MBPbIqV92AaeXatLxBI9gBaebbnrfifHhDYfgasaacH8akY=wiFfYdH8Gipec8Eeeu0xXdbba9frFj0=OqFfea0dXdd9vqai=hGuQ8kuc9pgc9s8qqaq=dirpe0xb9q8qiLsFr0=vr0=vr0dc8meaabaqaciaacaGaaeqabaqabeGadaaakeaat0uy0HwzTfgDPnwy1egaryqtHrhAL1wy0L2yHvdaiqaacqWFqessaaa@3768@⟩. Therefore, RAId_DbS will leave the number of peaks of intensity one in the processed data as a parameter determined by the S_u s_= ⟨ln *I*⟩. Note that in addition to its dependence on the spectrum considered, S_u _may also depend on the database used. Thus the statistics provided by Eq. (17) will be spectrum-specific and may also be database-specific. Once the number of intensity one peaks is fixed, one may continue to compute the second and third cumulants of the ln *I *distribution from the processed spectrum. The constants *β *and ⟨*x*^2^⟩ in Eq. (17) are thus fixed. Note that this procedure is applied regardless of whether the peak accuracy weight *w*_*i *_is turned on or off. However, when the number of theoretical peaks are variable, such as in the case of limiting only the molecular weights to be in a certain range, RAId_DbS treats both *β *and *γ *≡ *n/*(6⟨*x*^2⟩ ^*β*^2^) as two additional variables to be determined from fitting the score histogram.

RAId_DbS integrates the theoretical pdf, obtained from fitting score histogram with Eq. (17), from the high-scoring end down in order to obtain the *P*-value *P*(S) for score S. The *E*-value for a peptide with score S is then obtained by multiplying the *P*(S) by the effective database size. RAId_DbS uses the following method to estimate effective database size. Define

Nc(k)≡∑k′=0kUc(k′)andNn(k)≡∑k′=0k[Uc(k′)+U˜n(k′)].
 MathType@MTEF@5@5@+=feaafiart1ev1aaatCvAUfKttLearuWrP9MDH5MBPbIqV92AaeXatLxBI9gBaebbnrfifHhDYfgasaacH8akY=wiFfYdH8Gipec8Eeeu0xXdbba9frFj0=OqFfea0dXdd9vqai=hGuQ8kuc9pgc9s8qqaq=dirpe0xb9q8qiLsFr0=vr0=vr0dc8meaabaqaciaacaGaaeqabaqabeGadaaakeaafaqadeGadaaabaGaemOta40aaSbaaSqaaiabdogaJbqabaGccqGGOaakcqWGRbWAcqGGPaqkaeaacqGHHjIUaeaafaqabeqacaaabaWaaabCaeaacqWGvbqvdaWgaaWcbaGaem4yamgabeaakiabcIcaOiqbdUgaRzaafaGaeiykaKcaleaacuWGRbWAgaqbaiabg2da9iabicdaWaqaaiabdUgaRbqdcqGHris5aaGcbaGaeeyyaeMaeeOBa4Maeeizaqgaaaqaaiabd6eaonaaBaaaleaacqWGUbGBaeqaaOGaeiikaGIaem4AaSMaeiykaKcabaGaeyyyIOlabaWaaabCaeaadaWadaqaaiabdwfavnaaBaaaleaacqWGJbWyaeqaaOGaeiikaGIafm4AaSMbauaacqGGPaqkcqGHRaWkcuWGvbqvgaacamaaBaaaleaacqWGUbGBaeqaaOGaeiikaGIafm4AaSMbauaacqGGPaqkaiaawUfacaGLDbaaaSqaaiqbdUgaRzaafaGaeyypa0JaeGimaadabaGaem4AaSganiabggHiLdGccqGGUaGlaaaaaa@6357@

A peptide with correct N-terminal cleavage and with *k *miscleavages will be assigned an effective database *N*_*c*_(*k*). Similarly, a peptide with NNTC and with *k *miscleavages will be assigned an effective database size *N*_*n*_(*k*).

## Reviewers' comments

### Reviewer's report 1, first review comments

sent to the reviewers on July 26, 2007. Review received on September 11th, 2007.

Review by Wong Wing Cheong and Frank Eisenhaber, Bioinformatics Institute, Agency for Science, Technology and Research (A*STAR)

Matching of measured MS/MS spectra with theoretically calculated spectra for database peptides is the standard procedure for interpreting mass spectrometric data from protein samples. Alves et al. propose an alternative procedure for calculating *E*-values for peptide matches based on the expansion of the central limit theorem up to the third moment (skewness; not new though but with an elegant analytical derivation) to better fit the score distribution of a query spectrum.

General comments:

1) It should be noted that all methods that calculate scores and e-values with the actual intensities (here: *I*_*i *_in equation 1) suffer from the inaccuracies in the intensity measurements. The fragmentation and ionization efficiency for each peptide species given the same concentration can be very different from one another in terms of abundance in fragment ions. So it is difficult to decide whether the actual skewness in match scores' distribution is due to its natural form or due to insufficient sampling. To attempt to model skewness can be a double-edged sword in the sense that true hits for weaker spectra (for example, very incomplete b-y ion series) might be given higher p-values and marked as insignificant. As a result, one might lose true hits as false negatives though more noise spectra will also be thrown out. This effect should be examined more closely.

2) The existing methods are generally well performing for the identification of peptide hits in the case of spectra with sufficient clear signals. New methods can gain recognition in the field only for their ability to find significant hits even in more noisy or scarce spectra, not just for analytical elegance. For example, it was shown by Mujezinovic et al. (2006 Proteomics 6, 5117) show that the scores of hits dramatically improve if the amount of noise is reduced. The authors of this work attempt to demonstrate the performance of RAId_DbS to be more superior to others in a comparison but fall short of proving his point. Figures 2 and 3 do not provide the necessary arguments

3) In Fig. 2, the *E*-values of the various programs are directly compared, although the authors have previously declared that the *E*-values have to be transformed for this purpose (page 3 second paragraph). Thus, the figure is misleading. Curve clustering suggests that all the older methods calculate one kind of *E*-value whereas RAId_DbS belongs to another class of methods. Further, it would be sensible to consider only the reasonable *E*-value range << 1. Instead of using the nr-CR as described on page 11, it would be more appropriate to use nr with 90% or 80% similarity thresholds since there are many different classes of sequentially similar entries.

4) Figure 3 does not represent a true ROC curve. The absolute numbers of true and false positives appear to be calculated either with method-specific score or with method-specific *E*-value thresholds (note that SEQUEST does not provide *E*-values!!). One would expect the axes to show sensitivity (= TP/(TP + FN)) and specificity (= TN/(TN + FP)) instead. It should be possible to calculate and compare the area below the curves. For example at first glance, X!Tandem seems much inferior to RAId_DbS but when we look at Figure 3, their sensitivity and specificity performance appears quite similar. Especially in the practically important region of small false-positive assignments, the curves essentially overlay.

Minor

a) Will the newly derived pdf works better than other existing distributions (for example, the gamma distribution that can accommodate skewness)? With only a few measurements available, it is not clear how to decide what the naturally underlying distribution is in the far tail region. Understandably, the increase of the number of fitting parameters will improve the match with real score distributions.

b) The authors say that Fig. 1 was generated from a randomly picked spectrum. It appears rather that this spectrum is especially clean with large, almost complete b- and y-ion rows. To obtain an almost ideal fit of the score histogram and the fitted pdf is not a great surprise.

c) Summary: It would be advisable to provide a WWW-site for download of data and programs.

d) page 3, last paragraph: Please provide accession numbers instead of identifiers since only the latter are stable in time.

e) page 5, second paragraph: The weighting factors are purely heuristic without any fundamental justification.

f) page 5, penultimate paragraph: It is not good practice to refer to equation 17 after equation 1. This equation should be number 2.

g) page 6, 1st paragraph, equation (2) : This equation appears derived from a binomial distribution. Its information value at this point is unclear?!

h) page 14, reference list: It is not a good practice to refer to yet unpublished work; here, the authors have done it even twice.

#### Author's response to first review comments

Response and revised manuscript sent back to reviewers on September 17, 2007.

1) We believe there might be some misunderstanding here. First, we don't model the skewness as the skewness in the final score distribution is not determined based on the intensity profile of a given MS/MS spectrum. Note that the number of unit intensity peaks is used as a fitting parameter allowing for dynamic adjustment of the size of the skewness. We extended the central limit theorem to accommodate the case of small sample size. If the score distribution turned out to be symmetric, in our protocol, the skewness parameter *β *will have zero value as the best fit. That is, the statistics used in our method is intrinsically spectrum-specific, designed to take into account those effects mentioned by the reviewers. Regarding spectra of weak signals, it has been the major challenge of the field to extract the true hits from them. Our current method does not guarantee a better extraction, however, it will provide us with faithful *E*-values for each peptide hit given the main information content (b- and y-peaks) present in those spectra.

2) The fundamental reason to develop RAId_DbS is not just to develop another MS/MS database search tool. Rather, we want to provide a method capable of assigning correct *E*-values. The other existing methods might be well performing, but may not have accurate enough or even do not have *E*-value assignments. Fig. 2 demonstrates the *E*-value deviations of other search methods. Fig. 3 shows that RAId_DbS does not perform worse than other methods on top of having accurate *E*-value assignment.

3) The main point of Figure two is not to decide whether the *E*-values found among different methods are similar or not, instead, we are asking whether the *E*-value found by various methods are accurate or not. The standard definition of *E*-value gives rise to the theoretical curve in the figure. Agreement with the theoretical curve indicates faithful *E*-value assignment. Disagreement with the theoretical curve indicates inaccurate *E*-values. Since the number of spectra tested is only of order 10,000, one can't assess the *E*-value accuracy in the range of *E <*10^-4^. However, it is still possible to test the *E*-value range with *E > *10^-4 ^and *E <*10^2^. The large *E*-value portion is of course of low practical interest for reporting results from a single spectrum, but it does show the level of consistency between the reported *E*-values and the standard definition of *E*-value. This becomes particularly important when one needs to pool the results from various spectra together. Regarding the use of a database with similar entries purged, it is indeed a good suggestion for it mimicks better a random database. However, we find removing entries similar to target proteins sufficient to support our theoretical statistics, providing a larger range of usability of our statistics in real applications.

4) To emphasize the small false-positive assignment region, we replotted the ROC curves with the number of false positives plotted in log-scale while the number of true positives plotted in the linear-scale. Note that the ROC curve is a parametric plot of false positives versus true positives. The internal parameter used can be either the *E*-value or any monotonic function of the *E*-value. For methods not reporting *E*-values but scores, one assumes the score to be a monotonic function of the *E*-value and use the score as the internal parameter accordingly. Per the request of the reviewers, we have added the specificity versus sensitivity plots in Figure 3. Contrary to what the reviewers anticipated, we find that there is an even larger difference in performance between RAId_DbS and X! Tandem. In the figure legend, we also explain why this magnified difference may be an artifact due to low number of negatives reported by a specific method.

a) We anticipated Gaussian-type of distribution from our scoring construction. Theoretically speaking, if the number of theoretical peaks is large enough and the spectrum contains enough information, the score distribution will be very close to a Gaussian. The gamma distribution, although accommodates skewness, decaying with *e*^-*S *^does not have the right tail. Our pdf, on the other hand, naturally interpolate (see our theory section) between the Gaussian and the case of finite skewness.

b) We are afraid there might be some misunderstanding here. Looking at the score S, corresponding to maximum pdf in the plot, we find *S *~ 1. This implies that the majority of peptide entering the score histogram, if they are to have a complete b- and y-peaks present in the spectrum, will have peak intensity of only 2.718. Thus, it is highly unlikely that the spectrum has a complete b- and y- series for the candidate peptides. We had looked into a large number of score histograms from scoring various spectra, and all of them show excellent theoretical fits. Besides, if what is shown in Fig. 1 is a single best example, we won't be able to obtain *E*-value that traces theoretical curve so well (see Fig. 2).

c) We are in the process of setting up the website for RAId_DbS that allows the users to test run a few data set of their own and provide the options for code download.

d) We have added the accession numbers accordingly.

e) That is correct. This heuristic is to encourage fragment peaks with better m/z matching rather than just picking the m/z peaks of the largest intensities within the mass errors.

f) We agree with the opinion. However, since eq. 17 is the final results after going through eq.3 to eq. 16, it is rather hard to make it eq. 2. We therefore choose to keep the text as is and added a short text to remind the readers that it is the final results in the theory section that we are referring to.

g) Eq. (2) is another heuristic introduced to handle the case when the information level is too low, say, when there are too few peaks. When the weighted average number of evidence peaks found in the candidate list is below 2, there is simply not enough information to distinguish them well. In that situation, we switch to eq. (2) to obtain conservative p-values

h) We have removed one reference to the future and updated the other one.

### Reviewer's report 1, second review comments

sent to reviewers on July 26, 2007. first review received on September 11th, 2007; first authors' response sent to the reviewers on September 17th; second review comments received on September 21st, 2007.

Please find attached the reply to your comments. Although we still have some reservations, we think that the MS has improved as a result of the revision, especially with regard to Figure 3. Therefore, we think that the MS might be published in Biology Direct given the comment on the "major challenge in MS" being reduced to *E*-value computation is removed. Other comments might be considered valuable by the authors, too.

1) There is no way around that any value calculated as a function of intensities in the MS/MS spectrum depends on the accuracy of the intensity measurements. Therefore, the proposed method possibly results in more accurate expectation values for sequence hits but the information in the underlying spectrum might be not fully reliable.

2) The authors claim that accurate *E*-value calculation is the major challenge in mass spectrometry. This is certainly not the case (see Hernandez et al. 2006 Mass. Spectrometry Rev. v. 25, 235–254), especially in the practically not relevant range of large *E*-values. At the same time, we acknowledge that the authors have provided an analytical method for calculating them. In our reply, we have discussed to which extent this specific finding might change the practice of protein mass spectrometry and we are in doubt.

3) It is obvious that *E*-values have been derived for many methods with less fundamental considerations and they were never thought to be more than a rough guide for assessing false positive numbers; here, the authors have made progress. Nevertheless, it remains to be seen to which extent the type of MS data (profile versus centroid) puts some of the methods into an inferior position. It would also be of interest to analyze reasons for the non-linearity of the RAId_DbS curve in the range of *E*-values at about 1 and below.

4) We appreciate that the authors have added true ROC plots. Again, the usage of profile data instead of centroid data might have put some algorithms into a difficult position since some operate only with a limited number of peaks for protein identification. Also, the great difference between the two plots (3c and 3d, which differ only in the data format and the size of the dataset) is not discussed. In the range of small scores, OMSSA is superior to RAId_DbS in 3d but much inferior in 3c.

f) Nevertheless, you force the reader to lookup the end of the article when he just as started reading it.

#### Author's response to second review comments

1) The reliability of the information contained in the spectrum may still be judged from the *correct E*-value reported for the best hit. If even the best hit has a poor *E*-value, it is most likely that the information content in the spectrum is hard to retrieve due to high noise level.

2) We thank the reviewer for acknowledging our progress in producing more accurate *E*-values in peptide search. The full power of having correct *E*-values indeed awaits to be seen. In anticipation that the issue of having correct statistics will become more and more important, we have added a section discussing the accuracy of fitting the score pdf in general and have put RAId_DbS to test in this regard.

3) From what we can see in this study, it seems that every method's detecting power increases when using the profile data. Perhaps the question one may ask is that how much can each individual method gain by using profile data. The developers of the search methods may wish to investigate this problem for their own sake. Regarding the minor horizontal leap in RAId_DbS's *E*-value near *E *= 1, it is mainly due to that RAId_DbS reports only the top 250 hits and this may cause a slight under representation of high *E*-value hits.

4) As we said earlier, in general each method gains some when using profile data. However, it is *not *guaranteed that the gain will be of similar size. Consequently wishing to observe a consistent performance across different data type may not be a correct anticipation.

f) We must apologize for the inconvenience that this may have caused the readers.

### Reviewer's report 2

sent to the editorial board member/reviewer on July 23rd, 2007. Review received on August 15th, 2007. Review by Dongxiao Zhu (nominated by Arcady Mushegian) with contribution from Arcady Mushegian, Stowers Institute for Medical Research

1. While the aim of this paper is to derive score pdf for finite-size samples, it appears that infinite-size samples were assumed in eqs (7)-(9) and elsewhere. Please explain why this is so.

2. Is the result that the method performs when *y *is slightly more than 0, not only when *y *≫ 1, consistent with the expectations?

3. Page 6, second paragraph from bottom, possible definition error: the variance of a random variable is its second central moment, not the difference between first and second moment. Ibid., should it not be *y *instead of *y' *to be consistent to the rest of the article?

4. Authors may ve advised to keep closer to standard statistical representations in their derivation, for example, in the second paragraph from bottom, *E *[*X*^*k*^] is preferable as a definition of the kth moment of X, and *μ *should be used to represent population mean instead of X¯
 MathType@MTEF@5@5@+=feaafiart1ev1aaatCvAUfKttLearuWrP9MDH5MBPbIqV92AaeXatLxBI9gBaebbnrfifHhDYfgasaacH8akY=wiFfYdH8Gipec8Eeeu0xXdbba9frFj0=OqFfea0dXdd9vqai=hGuQ8kuc9pgc9s8qqaq=dirpe0xb9q8qiLsFr0=vr0=vr0dc8meaabaqaciaacaGaaeqabaqabeGadaaakeaacuWGybawgaqeaaaa@2DFD@. (The latter is usually used for representing sample mean NOT population mean, and may be confusing).

5. There is no particular reason to have supplementary materials for this paper: the article will be much easier to read if they were incorporated into the main text.

#### Author's response

1. We only assume that *n *is large, not infinite. Note that if n is infinite, the second moment ⟨*x*^2^⟩ will need to be infinite as well in order for (7)-(9) to make sense. And we apparently did not assume that.

2. Yes. As explained in the last paragraph of the theory section, the validity of the derivation is lost when 1 + 2*βy *becomes near zero. The accuracy of the derived expression, however, becomes better when *y *becomes larger. What is said there is that when *y *≫ 1, in the high scoring tail, the statistical significance assigned through our derived formula will work better.

3. We are sure it is a misunderstanding. The variance of a random variable is its second moment only if the random variable has zero mean. In general, the variance is the difference between the second moment and the second power of the first moment.

4. We thank the reviewer for the suggestion. However, we decide to keep our original notation since both type of notations are well accepted.

5. A very good suggestion indeed. We have moved the supplementary materials into the appendix of the paper to form a single article.

### Reviewer's report 3

sent to the reviewer on August 2nd, 2007.

Review received on October 2nd, 2007. Review by Shamil Sunyaev, Harvard Medical School

This manuscript introduces a new method for peptide identification for mass spectrometry proteomics. There is a growing need for more accurate computational methods for shotgun proteomics and the presented method has a high chance of becoming a popular tool in the field. The manuscript presents a derivation of an accurate p-value for the search using an asymptotic extension of the central limit theorem for the case of non-negligible skewness. Although the manuscript is technical in nature, due to the potential importance for applications, it fits Biology Direct. The manuscript is very well written and the presentation is clear.

## Authors' contributions

GA designed the research, wrote the software, carried out the research, and analyzed the results. AO helped in part of the software, carried out the research and analyzed the results. YKY derived the theoretical distribution, designed the research, analyzed the results and wrote the paper.
